# New insights into the evolution and local adaptation of the genus *Castanea* in east Asia

**DOI:** 10.1093/hr/uhae147

**Published:** 2024-05-28

**Authors:** Xinghua Nie, Yu Zhang, Shihui Chu, Wenjie Yu, Yang Liu, Boqian Yan, Shuqing Zhao, Wenli Gao, Chaoxin Li, Xueteng Shi, Ruijie Zheng, Kefeng Fang, Ling Qin, Yu Xing

**Affiliations:** Beijing Key Laboratory for Agricultural Application and New Technique, College of Plant Science and Technology, Beijing University of Agriculture, Beijing, 102206, China; Beijing Key Laboratory for Agricultural Application and New Technique, College of Plant Science and Technology, Beijing University of Agriculture, Beijing, 102206, China; Beijing Key Laboratory for Agricultural Application and New Technique, College of Plant Science and Technology, Beijing University of Agriculture, Beijing, 102206, China; Beijing Key Laboratory for Agricultural Application and New Technique, College of Plant Science and Technology, Beijing University of Agriculture, Beijing, 102206, China; Beijing Key Laboratory for Agricultural Application and New Technique, College of Plant Science and Technology, Beijing University of Agriculture, Beijing, 102206, China; Beijing Key Laboratory for Agricultural Application and New Technique, College of Plant Science and Technology, Beijing University of Agriculture, Beijing, 102206, China; Beijing Key Laboratory for Agricultural Application and New Technique, College of Plant Science and Technology, Beijing University of Agriculture, Beijing, 102206, China; Beijing Key Laboratory for Agricultural Application and New Technique, College of Plant Science and Technology, Beijing University of Agriculture, Beijing, 102206, China; Beijing Key Laboratory for Agricultural Application and New Technique, College of Plant Science and Technology, Beijing University of Agriculture, Beijing, 102206, China; College of Landscape Architecture, Beijing University of Agriculture, Beijing, 102206, China; Liaoning Economic Forest Research Institute, Liaoning Academy of Agricultural Sciences, Dalian, 116000, China; College of Landscape Architecture, Beijing University of Agriculture, Beijing, 102206, China; Beijing Key Laboratory for Agricultural Application and New Technique, College of Plant Science and Technology, Beijing University of Agriculture, Beijing, 102206, China; Beijing Key Laboratory for Agricultural Application and New Technique, College of Plant Science and Technology, Beijing University of Agriculture, Beijing, 102206, China

## Abstract

Chestnut plants (*Castanea*) are important nut fruit trees worldwide. However, little is known regarding the genetic relationship and evolutionary history of different species within the genus. How modern chestnut plants have developed local adaptation to various climates remains a mystery. The genomic data showed that *Castanea henryi* first diverged in the Oligocene ~31.56 million years ago, followed by *Castanea mollissima,* and the divergence between *Castanea seguinii* and *Castanea crenata* occurred in the mid-Miocene. Over the last 5 million years, the population of chestnut plants has continued to decline. A combination of selective sweep and environmental association studies was applied to investigate the genomic basis of chestnut adaptation to different climates. Twenty-two candidate genes were associated with temperature and precipitation*.* We also revealed the molecular mechanism by which *CmTOE1* interacts with *CmZFP8* and *CmGIS3* to promote the formation of non-glandular trichomes for adaptation to low temperature and high altitudes. We found a significant expansion of *CER1* genes in Chinese chestnut (*C. mollissima*) and verified the *CmERF48* regulation of *CmCER1.6* adaptation to drought environments. These results shed light on the East Asian chestnut plants as a monophyletic group that had completed interspecific differentiation in the Miocene, and provided candidate genes for future studies on adaptation to climate change in nut trees.

## Introduction

*Castanea* plants, known as ‘wood food’, are the earliest genus of Fagaceae developed and utilized by humans and play a unique role among nut trees in the world [1–3]. East Asia is the center of diversity of *Castanea* species [4], including four species: Chinese chestnut (*C. mollissima*), Henry chestnut (*C. henryi*), Seguin chestnut (*C. seguinii*) and Japanese chestnut (*C. crenata*). Among them, Chinese chestnut has a long usage history of >6000 years and is an important reference species for improving the quality of chestnut in the world [1, 5]. Interestingly, the Chinese chestnut is viewed as a potential resurgence solution for the nearly extinct American and European chestnuts, both of which have suffered severely from chestnut blight fungus (*Cryphonectria parasitica*) [6–8].

Several views and assumptions regarding the origin and evolution of *Castanea* are controversial. A previous study proposed the hypothesis that China is the origin center of *Castanea*, with the chestnut’s ancestry extending westward to Central Asia and then into the European Mediterranean, where it eventually evolved into the European chestnut. Meanwhile, the chestnut’s ancestors in north-east China and Japan evolved into the Japanese chestnut, then migrated across the Bering Strait to North America, and eventually evolved into the American chestnut [9]. However, another study, based on chloroplast DNA, considered that *C. crenata* is the most basal clade, forming sister lineages with other *Castanea* species, and that the American chestnut may have evolved from the European chestnut due to the separation of the continental plates of Europe and America [4, 10]. Despite different opinions on the origin of *Castanea*, East Asia is still recognized as one center of *Castanea* origin and has rich genetic diversity. Therefore, the East Asian chestnut species are dominant when studying the evolution and genetic relationships of *Castanea* plants.

The diverse habitats have also created different ecotypes of *Castanea* plants. Chinese chestnut was thought to have the strongest blight resistance and the widest natural range in China (distribution range: 21.5–41.5 N, 96.47–142.18 E) [1]. Chinese chestnut is the only *Castanea* plant in East Asia that has naturally spread north of the Qinling–Huaihe line, which is considered a dry/wet boundary between north and south China [11]. How Chinese chestnut adapted to the northern arid environment is crucial to the environmental adaptability of *Castanea* plants and the exploration of their excellent resistance genes. Changes in geographic environment and climate have profound effects on species populations and even species survival. Temperature and humidity are important factors that limit the natural spread of *Castanea*. The emergence of selection pressure gradually leads to changes in gene abundance, which in turn affects gene expression and changes in metabolites to improve environmental adaptability. The gene structure and the phenotype of the species have also changed accordingly and finally formed a dominant ecotype. Local adaptation is an indispensable part of the evolution of species, but at this stage many problems remain to be solved regarding the genetic basis and the evolutionary mechanism of their establishment and maintenance.

Due to the lack of sufficient genomic data, there are so many contradictions in previous studies that we know little about the evolution of modern *Castanea* plants. In the present study, we have provided some molecular evidence that *C. henryi* is the oldest species of the genus *Castanea* in East Asia and that the current distribution of *Castanea* species formed during the Miocene. Diverse natural environments shape different ecological types, so we focus on how chestnuts have adapted to different environments. The genomes of a wide collection of 394 chestnut accessions covering a wide range were analyzed. We determined that CmTOE1 can interact with CmZFP8 and CmGIS3 to promote the formation of non-glandular trichome, and then enhancing the environmental adaptability for chestnut. Finally, we identified some candidate genes associated with drought adaptation and confirmed that the interaction between *CmERF48* and *CmCER1.6* regulates the wax thickness of chestnut leaves. In summary, these studies were able to decipher the patterns of adaptation under natural selection. These variations provide a basis for a deeper understanding of the origin and evolutionary history of *Castanea* plants and reveal genetic loci related to their local adaptation.

## Results

### Genomic variation and genetic diversity of chestnut accessions in East Asia

**Figure 1:**
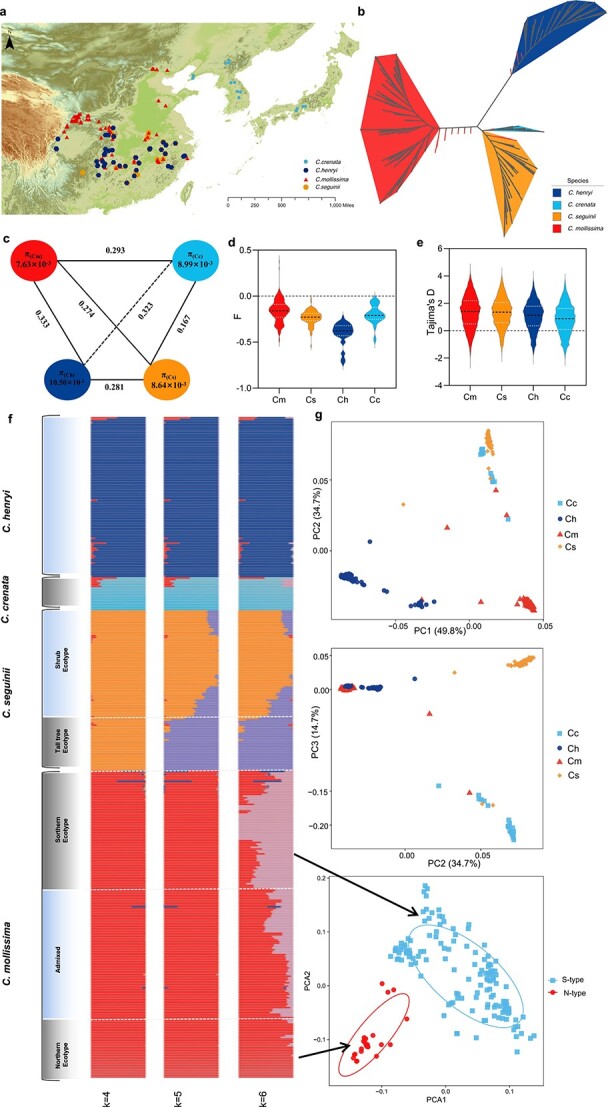
Collection, variation detection, clustering, and population structure of East Asian chestnuts. **a** Geographic distribution of the 394 materials on the map, including four species, displayed in different colors and shapes. **b** Unrooted phylogenetic tree analyses were performed on the genome-wide SNPs of 394 chestnut materials; the red lines represent interspecific mixed resources. **c** Nucleotide diversity (π) and population differentiation (*F*_ST_) of the four species. The value in each circle represents the level of nucleotide diversity (π) for each population, and the value on each line represents the population differentiation (*F*_ST_) for pairs of populations. **d**  *F* value of heterozygosity of four East Asian *Castanea* species. The smaller the *F*, the greater the heterozygosity. **e** Tajima’s *D* values of four East Asian *Castanea* species. **f** The population structure of 394 chestnut materials was displayed. When *k*=6, all samples have the best population division. **g** PCA plot of the 7.42 million whole-genome SNPs for 394 chestnut accessions. The arrows point to the distribution of two ecological types of Chinese chestnut, north type and south type, in PCA.

East Asia is recognized as the chestnut diversity center of the world, with varying environmental adaptability. To clarify the classification of East Asian chestnuts and examine the contribution of genome variation to environmental adaptability, a total of 394 chestnut individuals from East Asia were selected, comprising 166 *C. mollissima* (Cm), 100 *C. seguinii* (Cs), 108 *C. henryi* (Ch), and 20 *C. crenata* (Cc) trees (Fig. 1a, Supplementary Data Table S1 and Supplementary Data Figs S1 and S2). In total, we re-sequenced 2.27 Tb of data and mapped them to the reference genome (http://castaneadb.net). The results showed that the average sequencing depth was ~13.41× and the average mapping rate was 97.71%. A total of 7.42 million high-quality SNPs were identified, and the calculated density of all SNPs averaged one SNP per 95 bp. We found that 56.81% of SNPs are located in intergenic regions, 11.92% of SNPs are located in the intron region, and 3.66% of SNPs are located in the exon region (Supplementary Data Table S2). The non-synonymous/synonymous substitution rate of biallelic SNPs was 1.29.

To assess the genetic relationship and population structure characteristics of chestnut, we constructed an unrooted phylogenetic tree based on 53 346 four-fold degenerate synonymous sites (4DTv) (Fig. 1b, Supplementary Data Note S1). The results indicated that the chestnut plants could be classified into four categories strictly according to the species taxon; *C. henryi* was located at the base of the tree, hence *C. henryi* was the first to diverge from the ancestral species (Supplementary Data Fig. S3). The population differentiation (*F*_ST_) ranged from 0.167 to 0.323 (Fig. 1c), indicating that there was a high level of genetic differentiation between the four species. The nucleotide diversity (π) at the whole-genome level for all chestnut accessions was 8.94 × 10^−3^ and was higher than that of grape (π = 5.1 × 10^−3^), pear (π = 5.5 × 10^−3^), and apricot (π = 6.18 × 10^−3^) [12–14]. Of them, *C. henryi* has the highest nucleotide diversity (π = 1.05 × 10^−2^) compared with *C. seguinii* (π = 8.64 × 10^−3^) and *C. crenata* (π = 8.99 × 10^−3^). It is worth noting that *C. mollissima* was the most commonly and frequently collected, but its nucleic acid diversity was the lowest (π = 7.63 × 10^−3^) (Fig. 1c). The heterozygosity of the four species showed a consistent trend, heterozygosity ranging from low to high in the order *C. mollissima*, *C. crenata*, *C. seguinii*, and *C. henryi* (Fig. 1d). Linkage disequilibrium (LD) analysis showed that chestnut genomes exhibit relatively fast LD decays and the average *r*^2^ value was relatively low at ~0.185 (Supplementary Data Note S1, Supplementary Data Fig. S4). Tajima’s *D* values were all >0, *C. mollissima* having the largest value, ~1.34 (Fig. 1e).

Principal component analysis (PCA) clustering revealed all samples are mainly divided into four groups by species, with only a few individuals located between each group (Fig. 1g). This result was consistent with the result of *k* = 4 in the population structure analysis (Fig. 1f). In the stacked graph, we noticed that some chestnut accessions are mixed types. These showed that gene introgression is a widespread phenomenon in chestnut plants in nature. PCA results are also similar, particularly between *C. crenata* and *C. mollissima*, and there was a high proportion of mixed species between *C. mollissima* and *C. henryi* (Supplementary Data Fig. S5). At *k* = 5, the *C. seguinii* distributed in the Daba and Tianmu–Huangshan Mountains were independent in the *C. seguinii* population. Remarkably, the *C. seguinii* accessions in the Daba Mountains have higher tree potential and altitude distribution, have strong-quality timber, and reach even >15 m, which was not reported in previous studies. When *k* = 6, the *C. mollissima* accessions were divided into two clusters. Cluster 1 was a wild resource in the northern Qinling region (northern ecotype), and Cluster 2 was mainly a wild chestnut in the Tianmu Mountains (southern ecotype). This result supports the current opinion on the division of *C. mollissima* into two groups in China [15, 16]. There is obvious spatial distribution of the subspecies in chestnut, and we suspect that the diverse environmental factors, such as climate and geography, promote further differentiation of *C. mollissima*. What surprised us was that in addition to the Qinling Mountains and Daba Mountains, Tianmu Mountain may also have played an important role in the evolution and expansion of chestnut.

### Divergence patterns and demographic history of the East Asian chestnut

**Figure 2:**
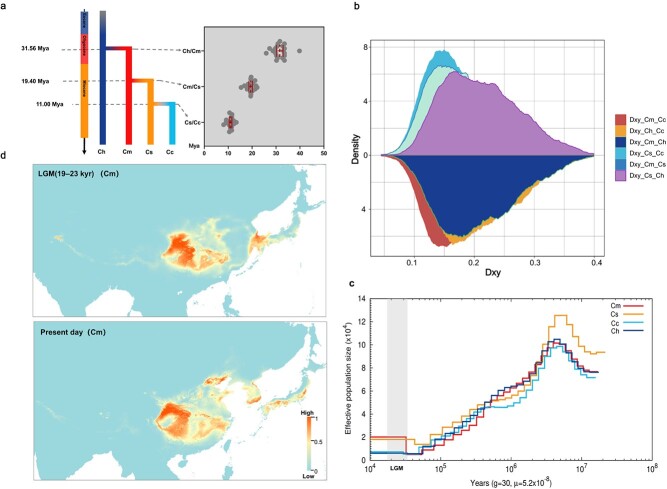
Speciation and demographic history of *Castanea* species. **a** Population split times among species using MSMC2 with *m* = 5.2 × 10^−8^ from estimates of pedunculate oak (*Quercus robur*) [74], and an average generation time of *g* = 30 years. Red bars indicate medians with 95% confidence intervals. **b** Frequency polygon of *D*_xy_ per base pair for each pair from the four taxa using a sliding window approach with a window size of 10 kb. **c** Demographic history of each species. The region shown in gray represents the LGM. **d** Prediction of the climate suitability of *C. mollissima* based on the Maxent model. The color scale shows the suitability rating; the climate data were sourced from http://www.worldclim.org/.

To date, the divergence events of chestnut plants are still unknown. Previous phylogenetic analysis based on chloroplast sequences indicated that the differentiation status of *C. mollissima*, *C. henryi*, and *C. crenata* was seriously inconsistent [10, 17]. In this study, we estimated the time of the most recent common ancestor of the four *Castanea* species using hidden Markov models in MSMC2. The result suggested that *C. henryi* first diverged in the Oligocene ~31.56 Mya. After this divergence, *C. mollissima* diverged from its Miocene ancestor at 19.40 Mya and *C. crenata* and *C. seguinii* at 11.00 Mya (Fig. 2a, Supplementary Data Fig. S6). Furthermore, by using 774 single-copy orthologous genes and a Fagaceae fossil (*Castanopsis*) as a correction point [18], the result was consistent with MSMC2 and showed that the four species represented a monophyletic-type family (Supplementary Data Fig. S7). The mean nucleotide difference (*D*_xy_) between different lineages showed that *C. seguinii* had the highest *D*_xy_ value with *C. henryi* at around 0.211, with the second highest *D*_xy_ value between *C. henryi* and *C. crenata* at around 0.210. The minimum *D*_xy_ value, ~0.175, was between *C. seguinii* and *C. crenata* (Fig. 2b). In summary, chestnuts completed differentiation within the genus in the Miocene. In the Miocene period, the altitude of the Qinling Mountains gradually rose, which had a significant impact on China’s climate and directly led to the formation of China’s north–south geography and climate [11, 19]. Remarkably, the geographic boundary coincided with the northern distribution limit of *C. seguinii* and *C. henryi*. We speculate that the uplift of the Qinling Mountains may have caused chestnut to diverge.

To better understand the population historical dynamics of chestnuts, pairwise sequentially Markovian coalescent (PSMC) analysis showed that four species experienced a continuous bottleneck effect that caused their effective population size to decrease since ~5 Mya (Fig. 2c). Near the Last Glacial Maximum (LGM; ~ 26 500 years ago), effective population sizes of four species recovered after the bottleneck. *C. mollissima* and *C. seguinii* had the highest contemporary effective population sizes (*N*_e_ ≈ 20 000). We then assessed the climatic suitability of *Castanea* using a maximum entropy model. The zone suitable for wild *C. mollissima* was the widest, and the highly adaptive survival zones were mainly Yanshan Mountain, Qinling Mountain, Wushan Mountain, Huangshan Mountain, Tianmu Mountain, and Daba Mountain. However, the adaptive survival zones for *C. henryi* and *C. seguinii* were mainly in the south of the middle and lower reaches of the Yangtze River. The highly adaptive survival zones for them were Huangshan Mountain, Wuyi Mountain, and Yandang Mountain. Notably, the highly adaptive survival zone of *C. henryi*, *C. mollissima*, and *C. seguinii* was mainly shared in the Qinling–Daba–Wushan Mountain and Tianmu Mountain. In addition, the land bridge between the Japanese islands, the Korean Peninsula and the Chinese mainland also provided a powerful condition for the continuous exchange of chestnut resources in the three places during the LGM (Fig. 2d, Supplementary Data Fig. S8). In summary, the Qinling–Daba–Wushan and Tianmu Mountains could represent either a hotspot of diversification or a glacial refuge. After the last Pleistocene interglacial period, chestnut plants gradually spread to the present-day area, which also supports the results of population division of the population structure.

### Accumulation of deleterious mutations in different chestnut species

**Figure 3:**
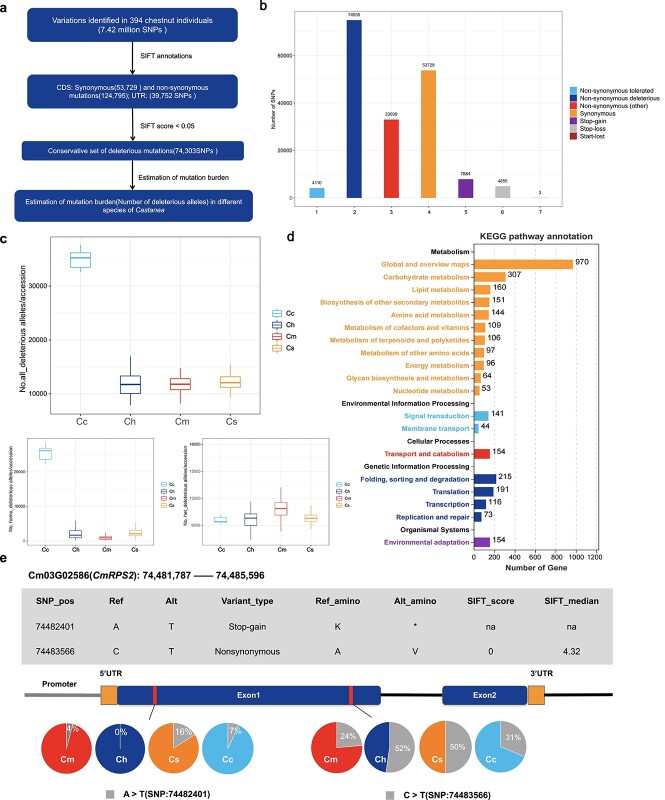
Deleterious variants in chestnut diversity panel. **a** The process of annotating deleterious mutations in this study. **b** Distribution of deleterious mutation sites in genomic regions. **c** Distribution of deleterious mutation sites among different chestnut species. **d** KEGG functional enrichment of 4842 genes involved in dSNPs. **e** Information on the deleterious mutation sites of *CmRPS2* (Cm03G02586) and their frequency distribution in four chestnut species.

To understand the effects of deleterious mutations on *Castanea* plants, we used SIFT software [20] to identify deleterious mutation features of the coding region across the genome using genetic variations and constructed a database of deleterious mutations. A total of 218 276 sites were identified in the coding region, of which 178 524 were in the coding sequence region, accounting for ~81.79%, and the rest were in the UTR region (Fig. 3a). Among all mutation sites, non-synonymous sites accounted for the largest proportion, at ~57.17%, followed by synonymous sites, at ~24.61%, and premature translation termination type sites accounted for only 5.84% (Fig. 3b). Those annotation sites with a SIFT score <0.05 are defined as conserved deleterious mutations (dSNPs), a score >0.05 is defined as tolerant mutations (tSNPs); a total of 74 303 dSNPs were predicted. These dSNPs involved 4842 genes, accounting for ~14.3% of the entire genome. Statistics on the accumulation of deleterious mutations in different species showed that *C. crenata* has the highest accumulation of deleterious mutation sites, especially homozygous deleterious mutations, which may be related to the narrow ecological range of *C. crenata*. However, *C. mollissima*, *C. henryi*, and *C. seguinii* accumulate fewer deleterious mutations, with *C. mollissima* having the fewest homozygous deleterious mutations and more heterozygous deleterious mutation sites (Fig. 3c).

Enrichment analysis was performed to understand the function of dSNPs involving 4842 genes. These genes were mainly enriched in metabolic pathways related to carbon metabolism, lipid metabolism, amino acid metabolism, and vitamin metabolism; genetic information processing related to spliceosome, mRNA surveillance, RNA degradation, protein processing in the endoplasmic reticulum, and ubiquitin-mediated proteolysis pathways; environmental adaptation related to plant–pathogen interaction, and circadian rhythm signaling pathways; and environmental information processing related to plant hormone signal transduction, ABC transporters, and phosphatidylinositol signaling system pathways (Fig. 3d). Disease resistance proteins containing the NB-ARC domain have been enriched in large numbers, including 35 members of the *RPS* gene family with numerous types of variation, such as *CmRPS2* (Cm03G02586) deleterious mutation site information, and two deleterious SNPs in exon 1 were predicted. There are significant differences in the frequency of mutation sites between different species, such as the mutation of site 74 483 566 on chromosome 3, which changes from C to T, resulting in the amino acid changing from A to V. This mutation site has the highest mutation frequency in the Henry chestnut population, accounting for ~52%, while it is only ~20% in *C. mollissima* and *C. seguinii* (Fig. 3e). These results suggest that deleterious mutations increase the genetic burden of chestnut adaptation to various biotic and abiotic stress environments and play an important role in the local adaptability and differences in resistance of the species.

### Genome-wide environmental association studies

**Figure 4:**
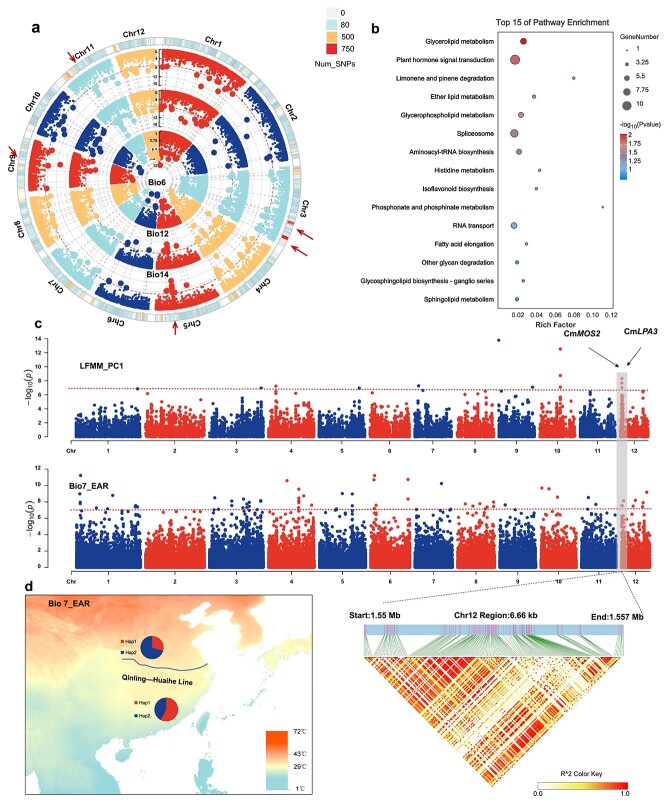
Genomic loci related to EVs. **a** Summarized genomic loci associated with EVs related to temperature and precipitation. Bio6, minimum temperature of coldest month; Bio12, annual precipitation; Bio14, precipitation of driest month. Red arrows indicate association hotspots with the environment. **b** Enrichment analysis of genes associated with more than five EVs. **c** Manhattan plots of SNPs associated with PC1 (LFMM) and Bio7 (EMMAX). The overlapped regions are highlighted using gray-shaded rectangles. **d** North–south distribution of different *CmLPA3* genotypes involved in local temperature and light adaptation on chromosome 12.

Many adaptive events in natural plant populations are polymorphic and then lead to numerous adaptations controlled by multiple genes [21, 22]. With the diversification of association analysis algorithms, various association methods have been put into practice at present to screen sites associated with local adaptability [22–26]. A total of 19 environmental variables (EVs) associated with plant survival were collected and genome-wide association analyses were performed (Fig. 4a). A total of 388 significantly associated SNP loci involving 1252 genes were identified (Supplementary Data Table S3) and 378 SNP loci were shared by different types of EVs, indicating that different EVs affect the same genome regions. A total of 88 loci were associated with at least five EVs, and these loci were significantly enriched in signaling pathway genes related to plant growth and development, stress, and response to the external environment, including glycerolipid metabolism, plant hormone signal transduction, spliceosome, aminoacyl-tRNA biosynthesis, response to stress, and defense response (Fig. 4b). In total, we found five association hotspots on chromosome 3 (two hotspots), chromosome 5 (one hotspot), chromosome 9 (one hotspot), and chromosome 11 (one hotspot) (Supplementary Data Table S4). Seven concatenated members of the zinc finger transcriptional regulator family on the top of chromosome 11 have been identified that play important roles in plant response to abiotic stress. For the three temperature-associated genomic hotspots, we found genes homologous to functional genes responsible for cold tolerance and salt stress in *Arabidopsis* (*LEA*) [27, 28] (Fig. 4c).

To address the high correlation between environmental factors and reduced redundancy, we performed a PCA analysis on 19 EVs to identify the principal component (PC) with the highest explanatory power. The analysis resulted in PC1, which effectively explained 54.49% of the total variation for all 19 EVs (Supplementary Data Fig. S9). We then analyzed the association features between PC1 and SNPs across the entire genome using LFMM and identified 57 significant association SNPs involving 205 genes. Of these genes, 22 genes overlapped with the 19 EVs (Supplementary Data Tables S5 and S6) and were mainly associated with environmental information processes such as cutin, suberin, and wax biosynthesis, flavonoid biosynthesis, and ABC transporter. Examples include a homologous gene, *CmANS* (Cm02G01635), in *Arabidopsis* associated with salt stress and strong light stress [29]; two tandem repeat genes *CmWDS1s* (Cm09G01917, Cm09G01918), showed that functional disruptions of these *Arabidopsis* homologs respond to drought stress in previous studies [30], one *Arabidopsis* homologous gene *CmMOS2* (Cm12G00146) associated with plant immunity [31]. The Qinling–Huaihe line in China represents a distinctive geographic boundary, dividing the country into two regions with distinct climate characteristics and markedly different temperatures throughout the year (Fig. 4d). Coincidentally, in this lineage only the wild Chinese chestnut among *Castanea* species can survive. We focused on the overlapping regions of the entire genome associated with LFMM and SNPs related to Bio7 (temperature annual range) to identify temperature-associated genes. One light and temperature-related gene and the *Arabidopsis* homolog gene *CmLPA3* (Cm12G00085) [32] was identified on chromosomes 12 (Fig. 4c). Among them, the Hap2 genotype of *CmLPA3* is the dominant genotype in the germplasm resources of northern wild chestnut, accounting for ~70.3%, while the Hap1 genotype is the dominant genotype in the germplasm resources of southern wild chestnut, accounting for ~57% (Fig. 4d, Supplementary Data Fig. S10).

### Selective sweep related to genetic differentiation of Seguin chestnut and local adaptation

Through long-term natural selection, local adaptations have gradually developed in the chestnut species. A typical case is a tall Seguin chestnut ecotype that we found in Shennongjia and that was adapted to high altitude. Tall Seguin chestnut is >15 m tall and common at altitudes of around 1800 m with lower temperatures and higher ultraviolet radiation. This is much higher than the range of the classical Seguin chestnut, which is a small tree or shrub that grows in hilly and mountainous areas [1]. Tall Seguin chestnut has more non-glandular trichomes on the leaf veins; these are specialized hair-like appendages that extend from the epidermal tissue of aerial plant parts [33]. It is often assumed that non-glandular trichomes create a natural physical barrier between the epidermal layer and the environment, thereby reducing water loss and excessive heat accumulation in plants, reducing frost damage, ultraviolet radiation, and mechanical damage, and reducing damage from harmful organisms [34].

**Figure 5:**
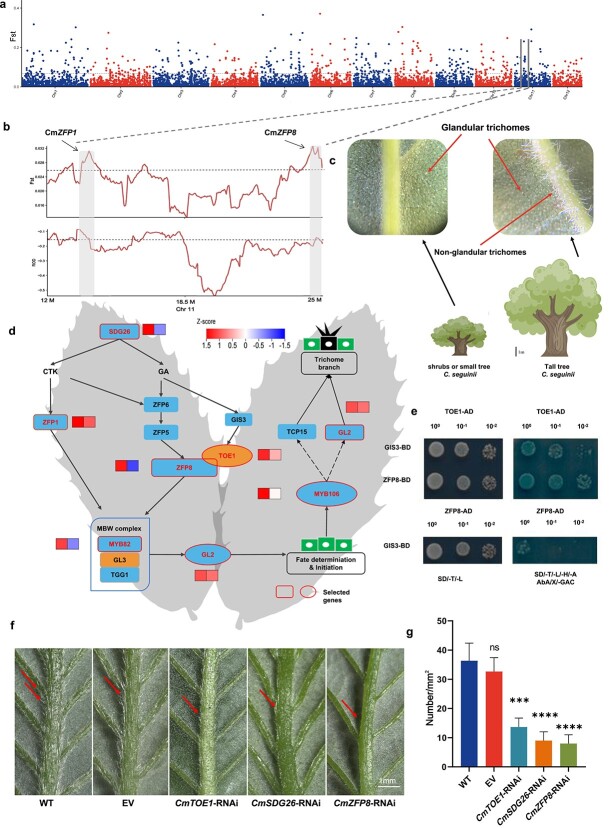
Genetic differentiation of Seguin chestnut and local adaptation. **a** Selective sweep for two ecotype populations of Seguin chestnut using *F*_ST_ (top 5%). **b** Selected genes related to the formation of non-glandular trichomes in 12–25 M of chromosome 11. **c** Differences in tree vigor and leaf back characteristics between two ecotypes of Seguin chestnut. **d** Schematic diagram of the formation of non-glandular trichomes; selected genes in tall Seguin chestnuts are indicated with red circles. **e** Verification of the interaction between ZFP8, GIS3, and TOE1, and between ZFP8 and GIS3. **f** Phenotypes of non-glandular trichomes on the back of the leaf of the transgenic tall Seguin chestnut (*CmTOE1*-RNAi, *CmSDG26*-RNAi, and *CmZFP8*-RNAi). **g** Density of non-glandular trichomes on the back of the leaf of the transgenic tall Seguin chestnut (*CmTOE1*-RNAi, *CmSDG26*-RNAi, and *CmZFP8*-RNAi). Scale bar = 1 mm. ^***^ represents *p *< 0.001; ^****^ represents *p* < 0.0001; ns represents no difference.

To investigate the environmental adaptability of tall Seguin chestnut, selective sweeps were performed for two ecotype populations (Fig. 5a and b, Supplementary Data Fig. S11). A total of 425 regions in populations of tall Seguin chestnut involving 501 genes were selected. Enrichment analysis involved pathways such as plant–pathogen interactions, RNA transport, nitrogen metabolism, plant hormone signaling, and anthocyanin biosynthesis. These genes played a key role in plant biotic and temperature stress, plant growth and development. It is worth noting that, in addition to tree vigor characteristics, we found significant differences in the presence or absence of non-glandular trichomes on the back of leaves between tall Seguin chestnut and classic Seguin chestnut. There may be defensive single hairs on the leaf veins of tall Seguin chestnut (Fig. 5c). It is noteworthy that six genes related to hair formation and development have been identified (Fig. 5d): *CmZEP1* (Cm11G00552), *CmZEP8* (Cm11G00954), *CmSDG26* (Cm01G02608), *CmMYB82* (Cm01G01374), *CmTOE1* (Cm11G02219), and *CmGL2* (Cm05G00390). All the above genes can be integrated into a pathway related to *CmSDG26* regulating the growth and development of *Arabidopsis* trichomes [35–37]. These genes may contribute to the formation of non-glandular trichomes in tall Seguin chestnuts to withstand the pressures of frost damage and ultraviolet radiation at high altitude. Interestingly, we also verified that TOE1 can interact with ZFP8 and GIS3 *in vitro*, while GIS3 cannot interact with ZFP8 (Fig. 5e). To investigate the function of the candidate genes in non-glandular trichome formation, we constructed RNAi vectors of *CmZFP8*, *CmTOE1*, and *CmSDG26* and then injected the vectors within *Agrobacterium* into the buds of the biennial branch of the tall Seguin chestnut. After 10 days, we compared the leaf non-glandular trichomes between the RNAi lines and the empty vector. The density of all non-glandular trichomes was significantly different between the empty vector and wild type, especially in the *CmZFP8*-RNAi line (Fig. 5f and g). This suggests that *CmZFP8* may play an important role in the formation of non-glandular trichomes in tall Seguin chestnut.

In addition, three tandem anthocyanin 5-*O*-glucosyltransferase genes (UGT75C1) have been identified on chromosome 6 that are related to anthocyanin synthesis and may also play a role in adaptation to cold environments. Simultaneously, large numbers of genes associated with auxin and gibberellin signals have also been identified (Supplementary Data Table S7), particularly the* SAUR* and *DELLA *family genes. These genes played an active role in the tree vigor difference of Seguin chestnut.

### Selection for drought resistance of wild Chinese chestnut in north and south China

**Figure 6:**
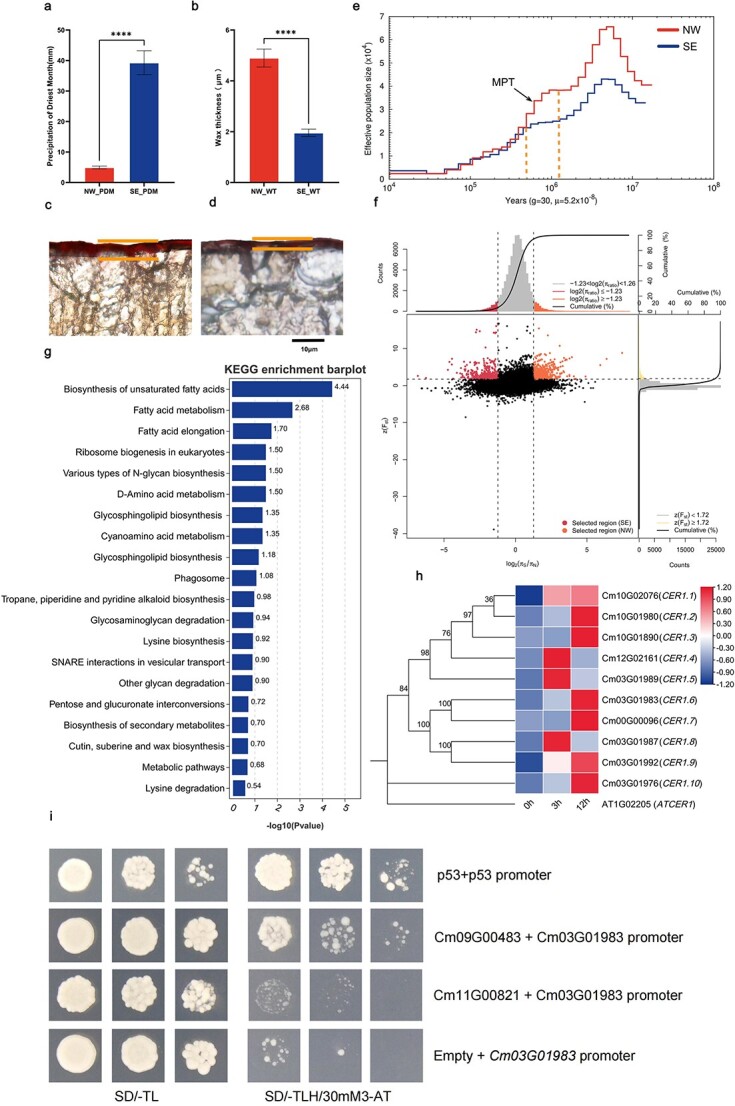
Genetics of drought resistance in the Chinese chestnut. **a** Precipitation of driest month for the NW and SE groups. **b** Analysis of variance of wax thickness for mature leaves in the NW and SE groups. **c** Schematic diagram of the wax layer of mature leaves in the NW group. **d** Schematic diagram of the wax layer of mature leaves in the SE group. **e** Demographic history in the NW and SE groups. **g** KEGG enrichment analysis of selected genes in the NW group. **f** Genome-wide distribution of selective sweep signals identified by π and *F*_ST_ of the NW and SE groups. **h** Phylogenetic relationships of *CER1*-homologous genes in Chinese chestnut. The heat map represents expression of 10 *CER1*-homologous genes in Chinese chestnut under drought treatment. **i** Verification of interaction between *CmERF48* (Cm09G00483) and the promoter of *CmCER1* (Cm03G01983) using a yeast one-hybrid assay.

The Chinese chestnut is the only species of *Castanea* that is common in northern and southern China and can adapt to more types of environments. To explore its environmental adaptability properties, we selected two groups with obvious environmental differences, the north-west (NW) group and the south-east (SE) group. It was also confirmed that these two groups belong to different subgroups in the population structure and cluster analysis. The NW group came from north-west China, a semi-arid area. The SE group came from south-east China where annual precipitation was three times that of the NW location (Fig. 6a, Supplementary Data Fig. S14). Because of the differences in precipitation between the two groups, the NW group is often affected by drought stress. Analysis of the leaf microstructure revealed that there was no significant difference in the thickness of the leaves between the two groups, but there was a significant difference in the thickness of the leaf wax layer (Fig. 6b–d, Supplementary Data Figs S15 and S16). The population history dynamics of the two wild ecotypes of Chinese chestnut showed that the effective population size of the NW population was significantly higher than that of the SE population around 500 000 years ago. However, the NW group experienced a rapid decline between 1.2 million and 500 000 years ago during the Middle Pleistocene Transition period (MPT), during which global ice content increased significantly, sea surface temperature decreased, and land desertification increased significantly (Fig. 6e). This climate characteristic resulted in a severe bottleneck effect in the NW group, and the population size in the NW group resource has declined to close to the SE group. The genomic composition of NW group was shaped by natural selection during the MPT.

Selective sweep was performed to identify genomic loci for local adaptation in both locations using the *F*_ST_ and π values of the two populations. A total of 1163 genes were identified in these candidate selection regions, comprising 702 genes in the NW group and 461 genes in the SE group (Fig. 6f). The results of enrichment analysis showed that there were significant differences in clustering between the two groups. The SE group was mainly enriched in the signaling pathways related to fatty acid elongation, zeatin biosynthesis, plant–pathogen interaction, and secondary metabolite biosynthesis. The NW group was significantly enriched mainly in the pathways related to unsaturated fatty acid biosynthesis, fatty acid metabolism and elongation, amino acid metabolism, various types of N-glycan biosynthesis, ribosome biogenesis in eukaryotes, and phagosome (Fig. 6g). The fatty acid elongation pathway was a significant pathway common to both groups (Supplementary Data Fig. S17). Further analysis revealed differences in the enriched genes, and a total of nine tandem repeat KCS (3-ketoacyl CoA synthase) genes were screened in the SE group. KCS also plays an important role in the interaction pathway between plant pathogens and plays an important role in allergic reactions and disease resistance in plants. However, in the NW group the *PPT*, *ELOVL4*, and *TER* genes are enriched. The specific locations of the pathway are completely different. In addition to the *KCS* genes, genes involved in plant–pathogen interaction, such as *FRK1*, *RPM1*, *FLS2*, *CNGC*, and *EIX1*, were also significantly enriched in the SE group. Of these, three genes encoding disease resistance proteins (*CmRPM1*: Cm10G01567, Cm10G01568, Cm10G01569) were selected; the defense responses of the homologs of these genes in rice and *Arabidopsis* against fungal disease have been verified [38–40].

Remarkably, we identified one *Arabidopsis* homologous *CER1* gene (Cm03G01983) and one *CER3* gene (Cm01G00496) in the two Chinese chestnut ecotypes. These genes are crucially involved in cutin and wax biosynthesis. The *CER1* gene encodes a decarbonylase that converts long-chain aldehydes into alkanes and is key to wax biosynthesis. Wax accumulation is associated with protection against water loss and contributes to drought tolerance of plants [41] and was upregulated by drought treatment, suggesting its role in the drought stress response. We have identified a total of 10 homologous genes of *CER1* in the whole Chinese chestnut genome, which is significantly expanded compared with *Arabidopsis thaliana*, *Fagus*, and *Quercus* (Supplementary Data Fig. S18)*.* The expression of 10 Chinese chestnut *CER1* homologous genes was upregulated during drought treatment by qRT–PCR (Fig. 6h). Meanwhile, we also identified four transcription factors associated with abiotic stress, including two *ERF* (Cm11G00821 and Cm09G00483) and two *SAGL1* (Cm07G00445 and Cm01G00902) homologous genes in *A. thaliana.* The *AP2*/*ERF* gene family plays an important role in plant stress. Previous studies in rice showed that *ERF71* can affect root structure and improve the drought tolerance of plants [42, 43]. Overexpression of *ERF38* in poplars could improve poplar tolerance to salt stress [44]. *SAGL1* encodes an F-box protein containing Kelch motifs and plays an important role in the regulation of plant growth and development, immune balance, and wax biosynthesis [45, 46]. To further investigate the relationship between these transcription factors and CERs, yeast one-hybrid experiments between pairs of these selected genes were performed. We verified the interaction between *CmERF48* (Cm09G00483) and *CmCER1.6* (Cm03G01983). There is no interaction between another *CmERF* (Cm11G00821) and *CmCER1.6* (Cm03G01983), between *CmSAGL1.1* (Cm07G00445) and *CmCER3* (Cm01G00496), between *CmSAGL1.2* (Cm01G00902) and *CmCER3* (Cm01G00496) (Fig. 6i, Supplementary Data Fig. S19). Regulation of plant wax layer accumulation by *ERF* transcription factors under drought stress offers a new perspective.

## Discussion

Species of the genus *Castanea* exhibit relatively little morphological variation. Their classification is based primarily on leaf morphology and variation in reproductive character states and cupule-to-fruit arrangement [18]. A general observation among Fagaceae is that sequence divergence between and within genera is very low, and pairwise sequence divergence values have ranged from about 0.1 to 1.3% [47]. In the early stages, high-throughput detection of nuclear genome variations in chestnut plants was very limited. Lang *et al*.’s results showed that *C. crenata* was independent of other East Asian *Castanea* plants and located at the base of phylogenetic trees using chloroplast sequences [10]. There was less agreement between chloroplasts and morphological characters in phylogenetic analysis. This study examined the evolutionary history of Asian *Castanea* plants by combining genomic analysis and archaeological evidence. The results of this study add more resolution to clarify that *C. henryi* is the earliest-divergent species of *Castanea* in East Asia, followed by *C. mollissima*, *C. seguinii*, and *C. crenata*. It is evident that the evolutionary relatedness of *Castanea* has been redefined, particularly the taxonomic position of *C. mollissima* and *C. crenata*. This contradiction of the phylogenetic tree, caused by the sequences of chloroplast and nucleus, is called the phenomenon of ‘nucleo-plasmic incongruence’, which presents a difficult problem in the study of molecular systems [48–52]. We speculate that the ancestral *Castanea* species has been preserved in the area that is the current habitat of *C. crenata*. Subsequently, at a certain point in time, the ancestor of *C. seguinii* heavily invaded a certain area occupied by *Castanea* plants. Since chloroplast genes are maternally inherited, the nuclear genes of *C. crenata*’s ancestor have been altered in a short time. The climate suitability of *Castanea* in East Asia also supports the existence of continuous adaptation regions between the Chinese mainland and the Japanese archipelago. In conclusion, we found that the monophyly of the genus *Castanea* is strongly supported by nuclear genes, and the Qinling–Dapa–Wushan region was the center of *Castanea* species diversity and then the center of diversity spread to the current area.

Trichomes are hair-like outgrowths of epidermal cells that cover the aerial parts of plants. Based on their structure and function, trichomes are classified as glandular or non-glandular trichomes, which can be unicellular or multicellular. Glandular trichomes play a crucial role in protecting plants from environmental stresses such as heat, low temperature, high UV radiation, and insect herbivory [53–56]. In East Asian chestnut plants, Henryi chestnut has no glandular trichomes, Chinese chestnut has evolved glandular trichomes to adapt to dry and cold environments. Classic Seguin chestnut has almost no glandular trichomes, while tall Seguin chestnut has more glandular trichomes to adapt to unique cold habitats. Japanese chestnut also has glandular trichomes, which may be related to their adaptation to cold environments. Based on the results of an *Arabidopsis* study, *SDG26* regulates cytokinin levels by affecting key rate-limiting enzymes such as *IPT1*, *IPT3*, and *IPT5*. Besides, *SDG26* regulates the expression of C2H2 transcription factors *ZFP1*, *ZFP6*, *ZFP8*, and *GIS*. The key activator of the trimeric complex MBW, including MYB23 and bHLH transcription factor *GL3* in the developmental process of trichome initiation, is regulated in part by *SDG26* [57]. Although we did not identify *IPT1*/*3*/*5* genes in the two groups between the tall Seguin chestnut and the classic Seguin chestnut, most of the homologous genes responsible for the formation of the *Arabidopsis* glandular trichome were swept. In contrast to *Arabidopsis*, *ZFP8* is homologous in the Seguin chestnut and we also identified a protein, TOE1, that interacts with ZFP8 in the cytokinin synthesis pathway. In the MBW complex, we did not identify a transcription factor homologous to MYB23, but identified another transcription factor of the MYB family, MYB82, which is also involved in the development of glandular trichomes in *Arabidopsis* [35]. This all suggests that while the formation of glandular trichomes in chestnuts follows a similar pathway to that in *Arabidopsis*, certain key genes are different. The relationship between glandular trichomes and environmental adaptability remains to be reported; the elucidation of the developmental pathway of glandular trichomes from chestnuts contributes to the ultimate analysis of the evolutionary status of glandular trichomes and also provides a guide to advance further investigation into resistance breeding in chestnut plants.

Drought has increasingly become a global problem. Thanks to the wide range of Chinese chestnut, it provides reliable materials for studying variation in drought resistance adaptation in different ecological niches. We scanned the drought-related selection traits and identified a large number of SNPs and candidate genes associated with drought in two populations of the north-western (semi-arid area) and south-eastern (humid area) groups, which experience markedly different precipitation. Our results also underscore the significant expansion of *CER1* in Chinese chestnut and the importance of wax biosynthesis for local adaptation under dry conditions. Apart from selective sweeps, this study also used environmental associations to uncover the genetic basis of chestnut plant adaptation. The rapid expansion of the *CER1* family in the Chinese chestnut is related not only to the thickening of the wax layer to adapt to the arid environment in northern China, but possibly also to the development of chestnut glandular trichomes [58]. In cucumbers, *CER1* can promote the formation of glandular trichomes [36]. Although we cannot confirm whether Chinese chestnut *CER1* is also required in the glandular trichome development in addition to wax layer synthesis, it is highly likely that Chinese chestnut has adapted to cold and dry environments by promoting the development of leaf appendages through the expansion of the *CER1* family.

In summary, this study provides new insights into the evolutionary history of the chestnut and how climate has shaped genomic characterization through natural selection. This will contribute to future work on resistance breeding.

## Materials and methods

### Sample sequencing

We conducted surveys in 17 provinces within China and collected the distribution of wild chestnut resources from Qingcheng Mountain, Sichuan Province (30.887578 N: 103.508105 E) in the west to Lin’an District, Zhejiang Province (30.334464 N: 119.520614 E) in the east, and from Maiji District, Gansu Province (34.390861 N: 106.033179 E) in the north to Hengyang, Hunan Province (26.871243 N: 112.352371 E) in the south. Japanese chestnut accessions were obtained from Liaoning Economic Forest Research Institute, which holds chestnut resources from North Korea, South Korea, and Japan. Genomic DNA was extracted from leaf samples using the CTAB method; 1.5 μg DNA per sample was used to prepare the libraries. Sequencing libraries were generated using a TruSeq Nano DNA HT Kit and whole-genome paired-end reads were generated using Illumina platforms.

### SNP calling and annotation

We performed quality control for raw resequencing data using fastp version 0.20 [59]. High-quality paired-end reads were mapped to the *C. mollissima* genome [60] using BWA version 0.7.12 [61]. PCR or optical duplicates were removed using SAMtools [62]. We performed SNP calling using a HaplotypeCaller approach as implemented in the package GATK version 3.8 [63]. Subsequently, further filtering of SNPs was performed with VCFtools using maf 0.05 || missing 0.2 || minDP 2 || maxDP 1000 || minQ 30 || minGQ 50. High-quality SNP annotation was performed using SnpEff version 5.1 [64].

### Phylogenetic tree and population structure

We first used the annotation results to screen for 53 346 four-fold degenerate sites (4DTv). Then, IQ-TREE version 2.0.3 [65] with maximum likelihood was used to build phylogenetic trees with *Castanopsis tibetana* as the outgroup. PCA was performed using PLINK version 1.90 [66]. VCFtools version 0.1.17 [67] was used to calculate *F*_ST_ and π with a 10-kb window. Correlation coefficients (*r*^2^) were calculated for all SNPs to measure LD decay using PopLDdecay v3.41 [68]. Subsequently, we screened a subset of biallelic and high-quality SNPs with a call rate ≥ 90%, MAF ≥ 5% and indep-pairwise 50 10 0.2. The population structure was calculated using ADMIXTURE version 1.3.0 [69] and cross-validation error was tested for each *k* value from 2 to 10 to identify the best genetic cluster *k*.

### Genetic diversity

Genetic nucleotide diversity π, Tajima’s *D*, and population differentiation statistics (fixation index, *F_ST_*) were calculated using VCFtools version 0.1.17 [67]. We utilized the script written by Simon Martin’s team (https://simonmartinlab.org/) to calculate the *F* value of heterozygosity and *D*_xy_ (population differentiation) of four East Asian *Castanea* species using a 100-kb non-overlapping window.

### Divergence events and demographic analyses

A total of 774 single-copy genes were used to build phylogenetic trees, and fossil *Castanopsis* plants as correction points [18] to estimate the differentiation time of *Castanea* plants using the RelTime-ML module. We also employed the relative cross-coalescence rate (RCCR) of MSMC2 [70, 71] to infer divergence time on two individuals (four haplotypes) for each species. Median population split times were deduced from the results of 20 random combinations for each comparison. We generated the diploid consensus sequence of each sample using SAMtools [62] to infer population size in PSMC [72]. For PSMC and MSMC2 analyses, we assumed an average mutation rate of *m* = 5.2 × 10^−8^ from estimates for pedunculate oak (*Quercus robur*) [73], and an average generation time of *g* = 30 years.

### Habitat suitability modeling

We used the maximum entropy model (Maxent) [74] to evaluate habitat suitability. Nineteen bioclimatic variables of the 2.5 arc-minute scale (CCSM4) data for 1970–2000 and the LGM from WorldClim 2 [75] were downloaded (http://www.worldclim.org/). We masked these climate layers to the area that was reasonably surveyed and removed highly correlated variables with a Pearson’s correlation coefficient ≥0.8. Data formats were transformed using ArcMap 10.8 (https://desktop.arcgis.com/en/arcmap). Ten replicates with cross-validation were performed for each analysis.

### Identifying deleterious mutations.

Amino acid substitution and the SIFT score database of four species were predicted and constructed using the SIFT algorithm [20]. We used *C. tibetana* as an outgroup and performed allele polarization based on the methods of a previous deleterious variation study [23]. Firstly, we discarded homozygous sites that were identical to *C. tibetana*. If the SNP genotype of a given sample was homozygous and different from *C. tibetana*, we determined that it was a homozygous deleterious SNP. If the SNP genotype of a given sample was heterozygous, we determined it as a heterozygous deleterious SNP.

### Genome-wide environmental association analyses

Genome-wide environmental association analyses (GWEAS) were performed with a set of 348 784 SNPs with MAF >0.05 and a data missing rate <0.5 using the mixed linear model (MLM) with EMMAX software [76]. The kinship matrix and PCA were used as the random effect and fixed effect covariates, respectively. The genome-wide significance threshold was set as 0.05/total number of SNPs [1.43E−7 (0.05/348784)] using Bonferroni correction. To more accurately identify environmental association loci, we removed redundancy from 19 environmental factors and performed PCA analysis on these environmental factors using Prism 9.0 [77]; the PCA result showed that the first environmental principal component (PC1) explained >50% of the total variance. Subsequently, PC1 was used to perform association analysis on genome-wide SNPs using the Late Factor Mixed Effect Model (LFMM) with the following parameters: -p 8 -K 3 -I 10000 [78].

### Selective sweep

We divided the intraspecific resources into ecological types according to the characteristics of resources and their ecological niche differences. We use multiple methods [*F*_ST_, π, and ROD (reduction of diversity)] to identify selected regions and genes in the genome using VCFtools with a 100-kb sliding window and a step size of 10 kb. We selected the top 5% of the regions as candidate regions. GO (Gene Ontology) and KEGG (Kyoto Encyclopedia of Genes and Genomes) enrichment analysis was performed using the OmicShare tools (https://www.omic  share.com/tools).

### Vector construction and transient transformation

Specific fragments of the *CmTOE1*, *CmSDG26*, and *CmZFP8* genes were cloned, which were 304, 399, and 282 bp, respectively. The specific fragments were connected to the PK7GWIWG2(II) RR-277 vector using the Gateway method and transformed into *Escherichia coli* (DH5α). We performed DNA sequencing, transformed the plasmids into *Agrobacterium tumefaciens* (GV3101), and stored at −80°C in a refrigerator.

To verify the effects of *CmTOE1*, *CmSDG26*, and *CmZFP8* on the density of non-glandular trichomes on the back of tall Seguin chestnut leaves, transient transformation of tender buds from Seguin chestnut branches was performed. The primers used are listed in Supplementary Data Table S8. Activated *A. tumefaciens* infection solution containing *CmTOE1*-RNAi, *CmSDG26*-RNAi, and *CmZFP8*-RNAi (10 mmol/l MgCl_2_, 10 mmol/l 2-morpholine ethanesulfonic acid, and 200 μMmol/l acetosyringone) was injected into the base of tender buds, which were incubated in a dark incubator at 28°C for 2 days. After the leaves were expanded, they were identified at the genomic and transcriptional levels. Subsequently, we used a microscope to record the non-glandular trichome status on the back of leaves and calculate the density of non-glandular trichomes (the number of non-glandular trichomes in a 1-mm^2^ visual field, repeated three times).

### RNA extraction and qRT–PCR analysis

Verification of candidate genes for drought response at 0, 1, 3, 6, 9, and 12 h with water loss drought treatment was performed on annual chestnut seedlings with three independent biological replicates for each. For verification of candidate genes for non-glandular hair formation, we used the mature leaves of tall Seguin chestnut and classic Seguin chestnut resources as experimental materials. These resources were grafted on Dalian Chestnut Resource Nursery (Liaoning Economic Forest Research Institute) in 2021. The total RNA of all samples was extracted using an E.Z.N.A.^®^ Plant RNA Kit (Omega, Guangzhou, China) and the quality of RNA was evaluated using a NanoDrop spectrophotometer (NanoDrop Technologies, Wilmington, DE, USA). Ten *CmCER1*s, *CmZEP1*, *CmZEP8*, *CmSDG26*, *CmMYB82*, *CmTOE1*, and *CmGL2* were analyzed by qRT–PCR. The data were normalized for the expression level of *CmActin* as the internal control.

### Statistical analysis

Differential analysis of the relative expression values from qRT–PCR and non-glandular trichome density among different samples was performed in GraphPad Prism 9.0 [77].

## Supplementary Material

Web_Material_uhae147

## Data Availability

The data supporting the findings of this work are available within the paper and its Supplementary Information files. A reporting summary for this article is available as a supplementary information file. The sequencing data in this study have been deposited in the NGDC Sequence Read Archive under accession number PRJCA017848 (https://ngdc.cncb.ac.cn/gsub/submit/bioproject/PRJCA017848). The datasets of 19 bioclimatic variables of the 2.5 arc-minute scale (CCSM4) data for 1970–2000 and Last Glacial Maximum (LGM) were obtained from WorldClim 2 [75] (http://www.worldclim.org/). The code for bioinformatics analysis is publicly available in the GitHub repository (https://github.com/niexinghua/Saner).
