# Supplementary material for: New insights into the evolution and local adaptation of the genus Castanea in east Asia
Source: Hortic Res. 2024 May 28;11(7):uhae147. doi: 10.1093/hr/uhae147 (PMC11233864; doi:10.1093/hr/uhae147)
Supplement: Web_Material_uhae147 [file web_material_uhae147.zip › Supplementary Information_R1.docx]

**Supplementary [Information](https://static-content.springer.com/esm/art:10.1038/s41467-018-07744-3/MediaObjects/41467_2018_7744_MOESM1_ESM.pdf)**

**Note S1 Phylogenetic analysis of the 394 samples along with *Castanea* plants in East Asia**

In order to more accurately explore the evolutionary relationship of *Castanea* plants in East Asia, 53,346 SNPS of 4dTV were used to construct a maximum likelihood phylogenetic tree (Fig. S3), and a material of *Castanopsis tibetana* were used as the outer group. The phylogenetic tree results showed that *Castanea* plants in East Asia were a typical single system, *C. henryi* was located at the base, and differentiated first, followed by *C. mollissima*, and *C. seguinii* and *C. crenata* finally differentiated. In terms of geographical distribution of chestnut plants, *C. henryi* and *C. seguinii* are only distributed in the middle and lower reaches of the Yangtze River and in the south, while *C. mollissima* crosses the Yangtze River and has a wider distribution range, there are the most diverse chestnut plants in South China. Therefore, we speculate that South China may be the origin center of *Castanea* in East Asia. In addition, it is worth noting that intraspecific materials are arranged according to geographical location. It can be seen from the *C. mollissima* that the materials from Shennongjia and the north are clustered in a group, and the materials from the Yangtze River basin and the south are clustered in a group. Therefore, Shennongjia maybe the center of genetic diversity of Chinese chestnut, and gradually evolved into the two major Chinese chestnut ecotypes with obvious character differences from north to south.

**Note S2 The characteristics of LD in Japanese chestnut**

The analysis of linkage disequilibrium of *Castanea*, showed that *Castanea* genomes have relatively short LD distances and relatively rapid LD decays (Fig. S4). This could be explained by high outcrossing rates that are maintained by self-incompatibility. In addition, we observed that the DNA decay of genome of Japanese chestnut was significantly slower than that of other *Castanea* plants. This feature may be closely related to the fact that the Japanese chestnut resources we collected were cultivated after the artificial intervention. Meanwhile, the recent inter-specific event provided more DNA fragments of Chinese chestnut for the genome of the tested Japanese chestnut materials, which officially increased the linkage of Japanese chestnut.


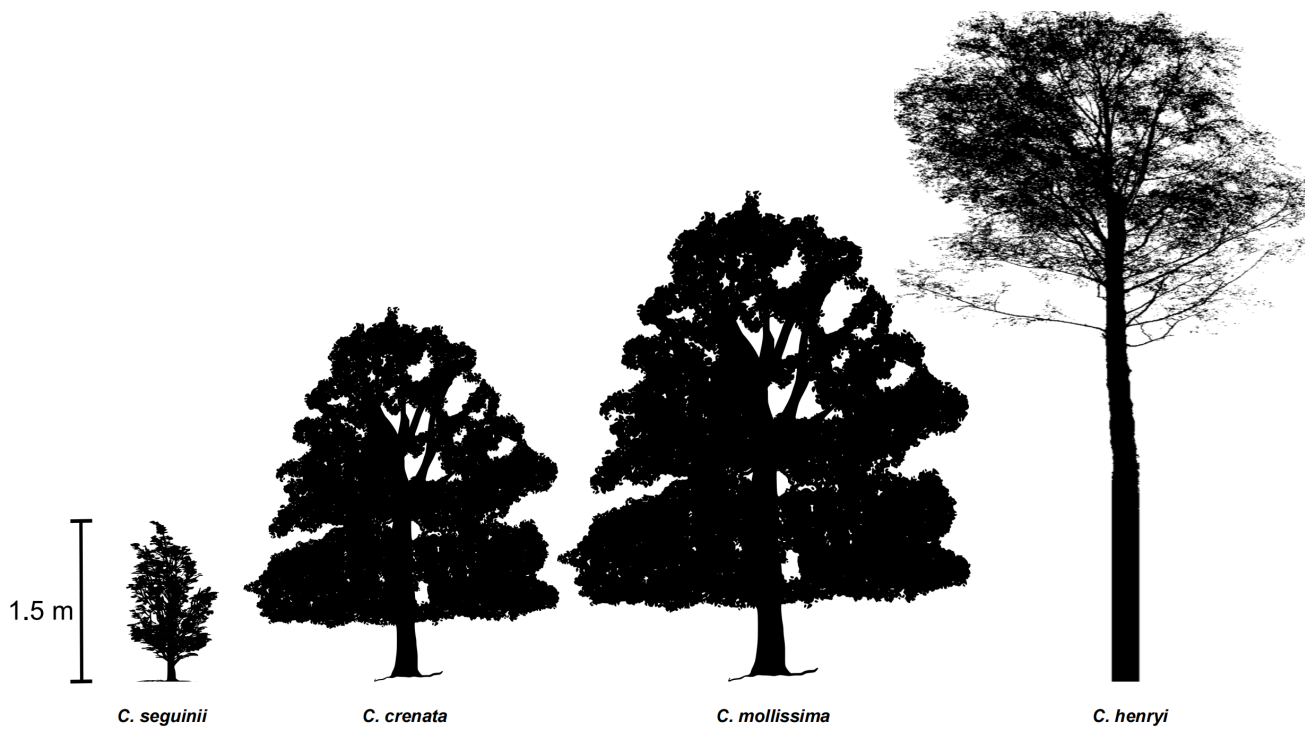


**Fig. S1** Schematic diagram of inter species tree vigor characteristics of *Castanea* plants. *C. seguinii* is often a shrub, with an adult tree of about 1.5-3 meters; *C. crenata* is often a tree, reaching a height of up to 15 meters; *C. mollissima* is often a tree, reaching a height of 15-20 meters; *C. henryi* is often large tree with strong upright characteristics and can reach a height of up to 30 meters.


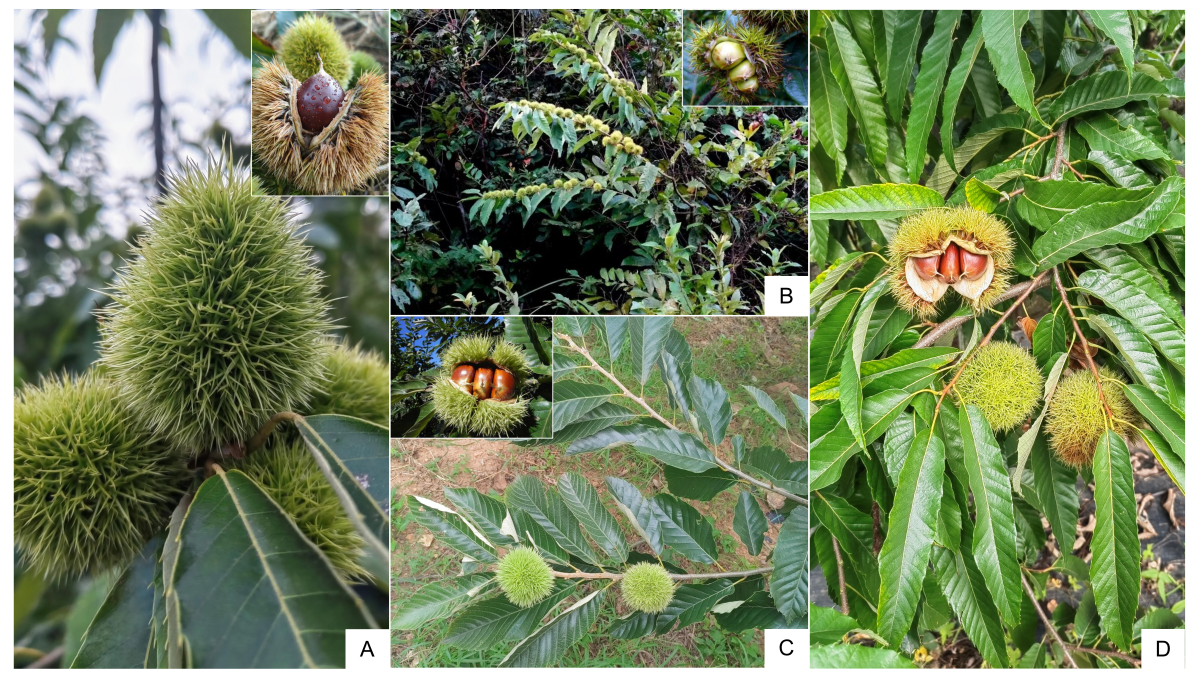


**Fig. S2** The fruiting habits and nut characteristics of *Castanea* plants. *C. henryi* has one nut per bud, while *C. seguinii*, *C. mollissima*, and *C. crenata* have three nuts per bud. *C. seguinii* has a unique habit of bearing fruit in clusters.


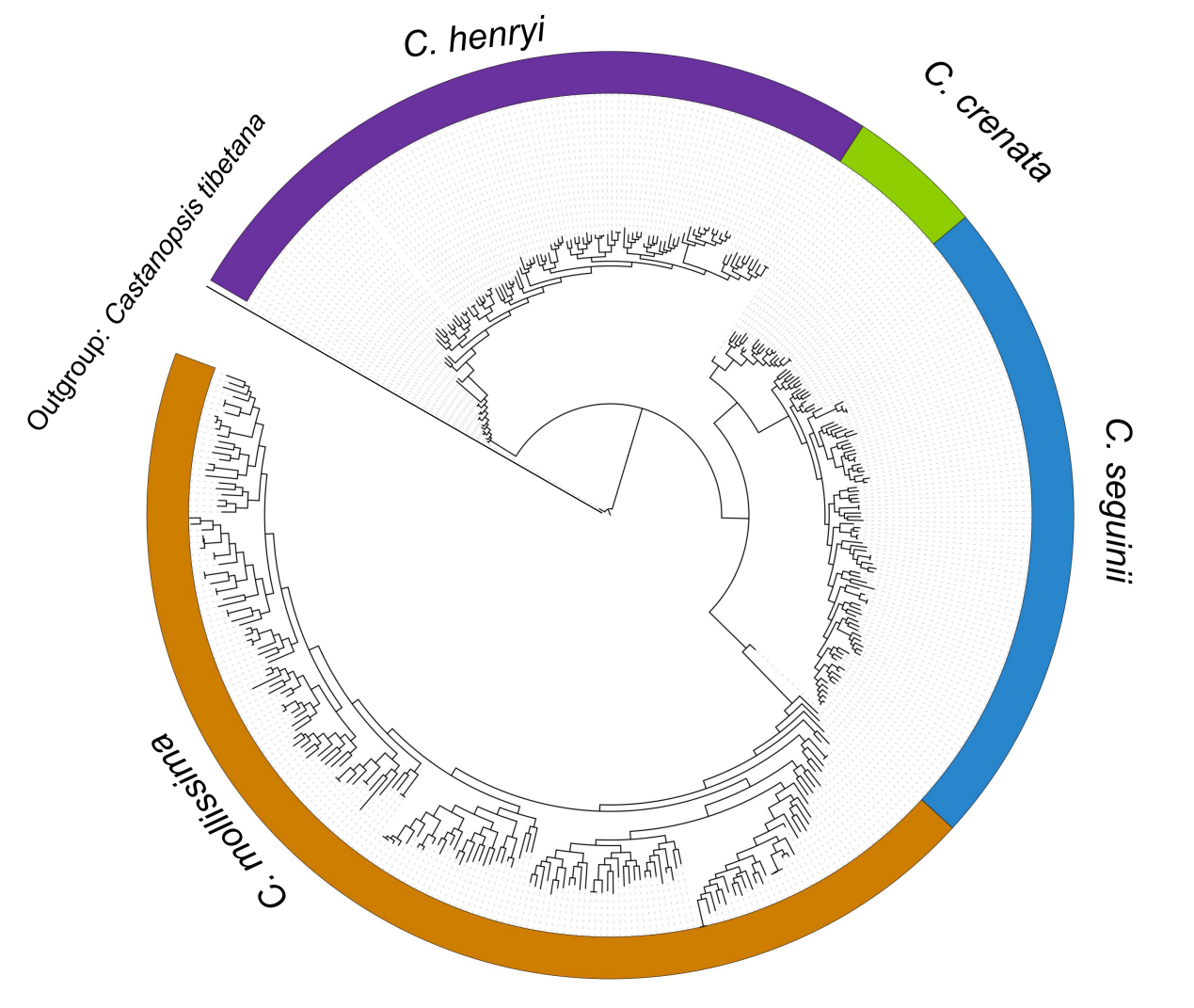


**Fig. S3** Maximum likelihood phylogenetic tree of 394 chestnut accessions with *Castanopsis tibetana* used as an outgroup. Purple represents *Castanea henryi*, green represents *Castanea crenata*, blue represents *Castanea seguinii*, and brown represents *Castanea mollissima*.


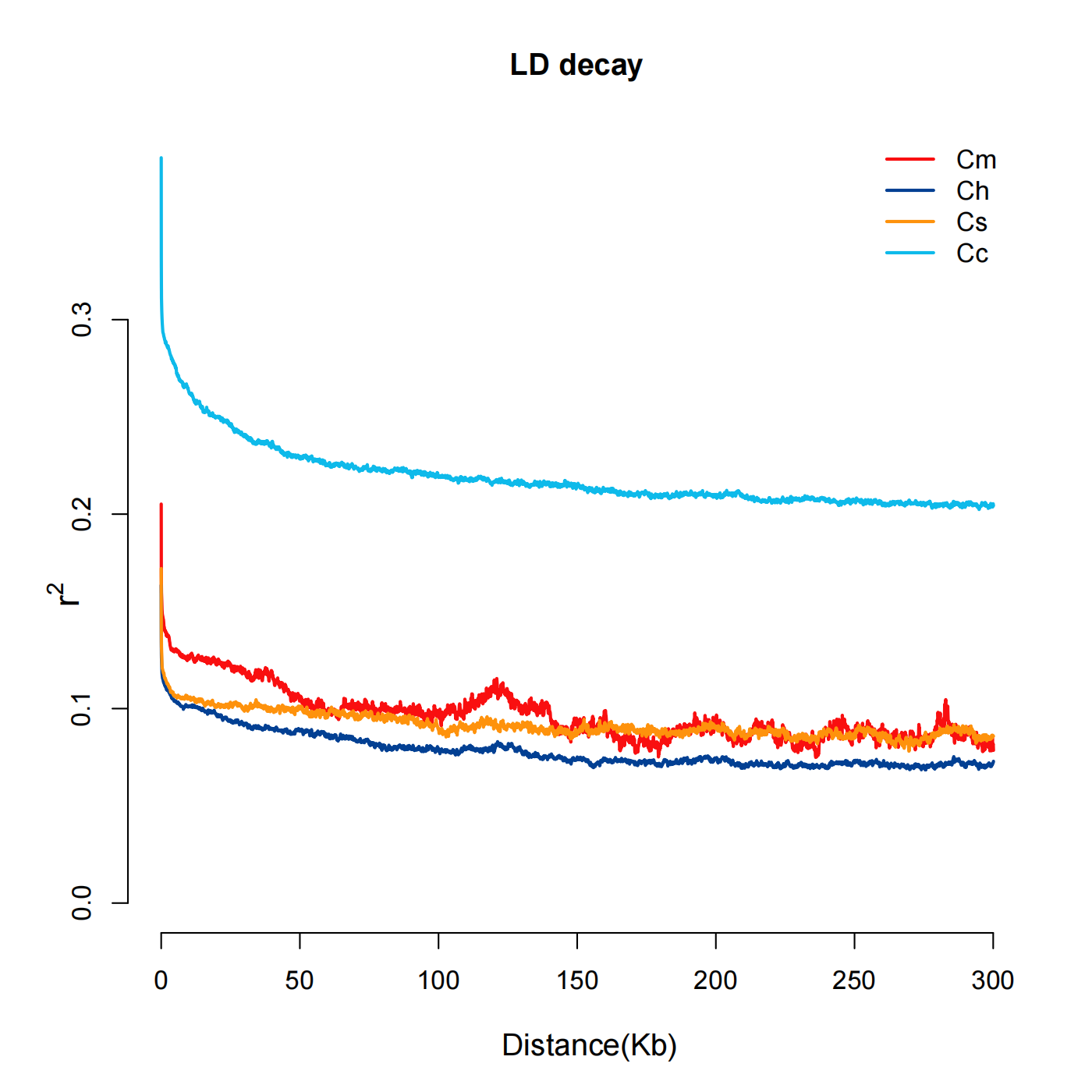


**Fig. S4** Linkage disequilibrium patterns of four typical species: *C. mollissima* (Cm), *C.* *seguinii* (Cs), *C. henryi* (Ch) and *C. crenata* (Cc) based on the SNPs from the accessions sequenced in this study.


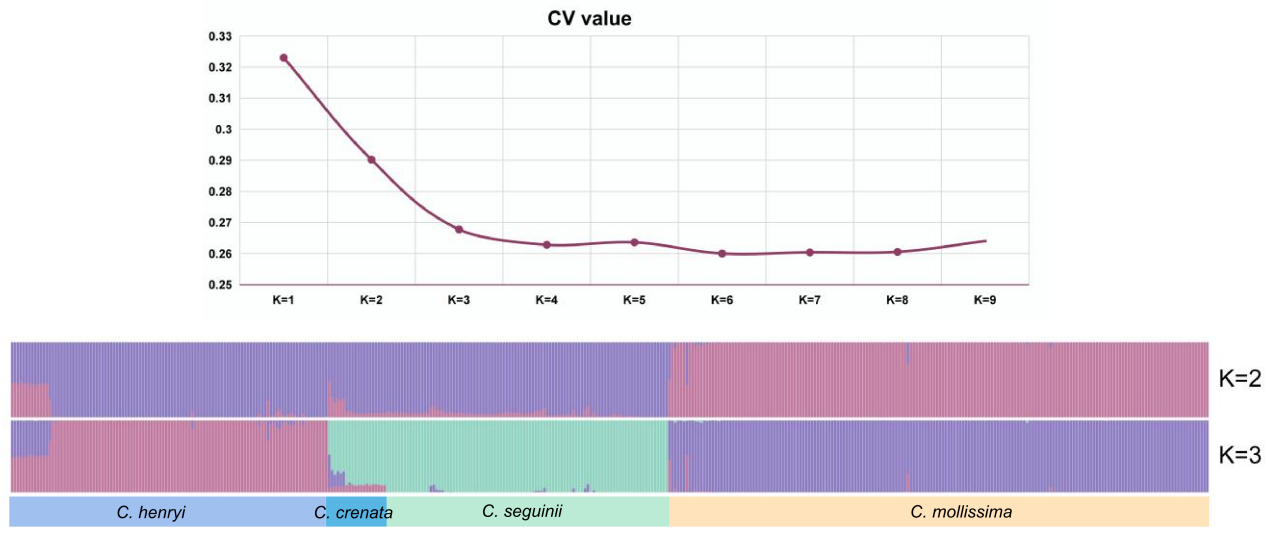


**Fig. S5** The population structure (CV value of K=1~9) of all 394 chestnut accessions inferred from whole-genome SNPs.


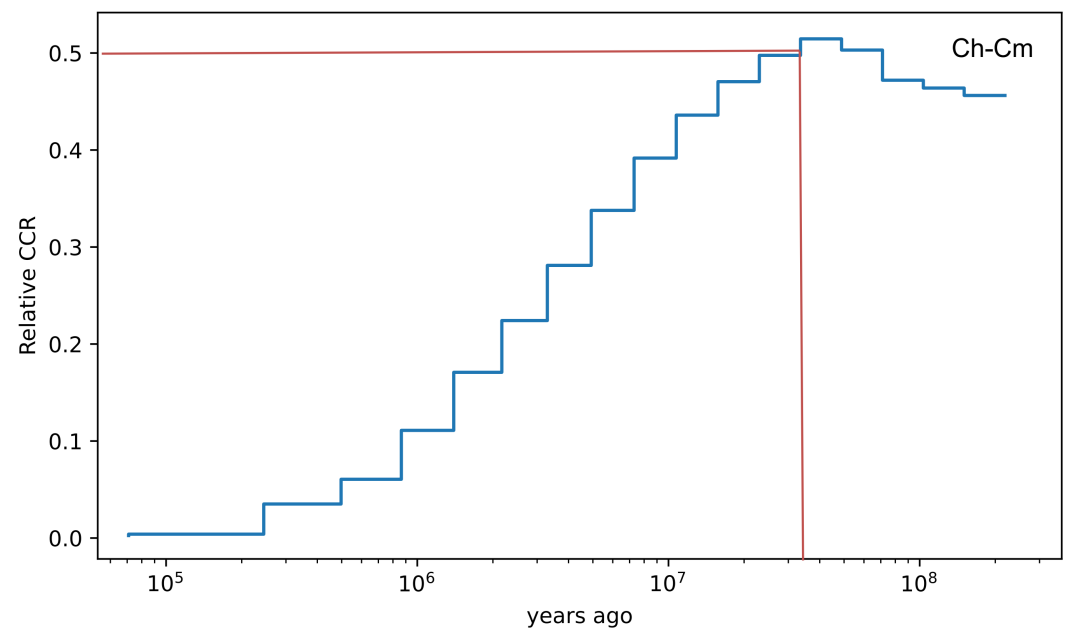

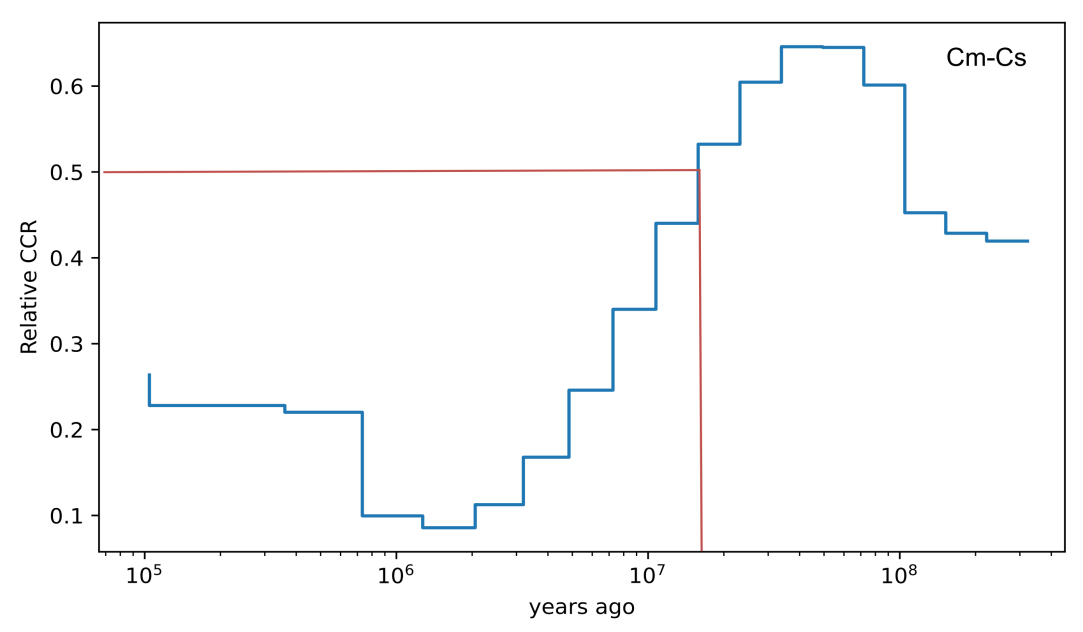


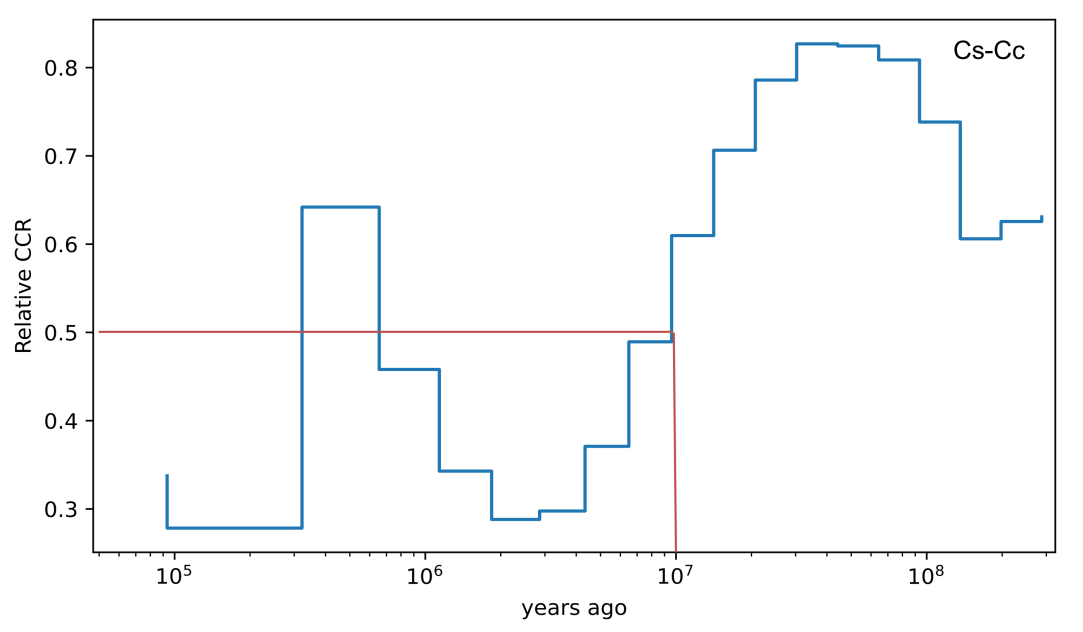


**Fig. S6** Estimated split times among four species with MSMC2. Ch-Cm(about 31,560,000 year ago), Cm-Cs(about 19,400,000 year ago) and Cs-Cc(about 11,000,000 year ago).


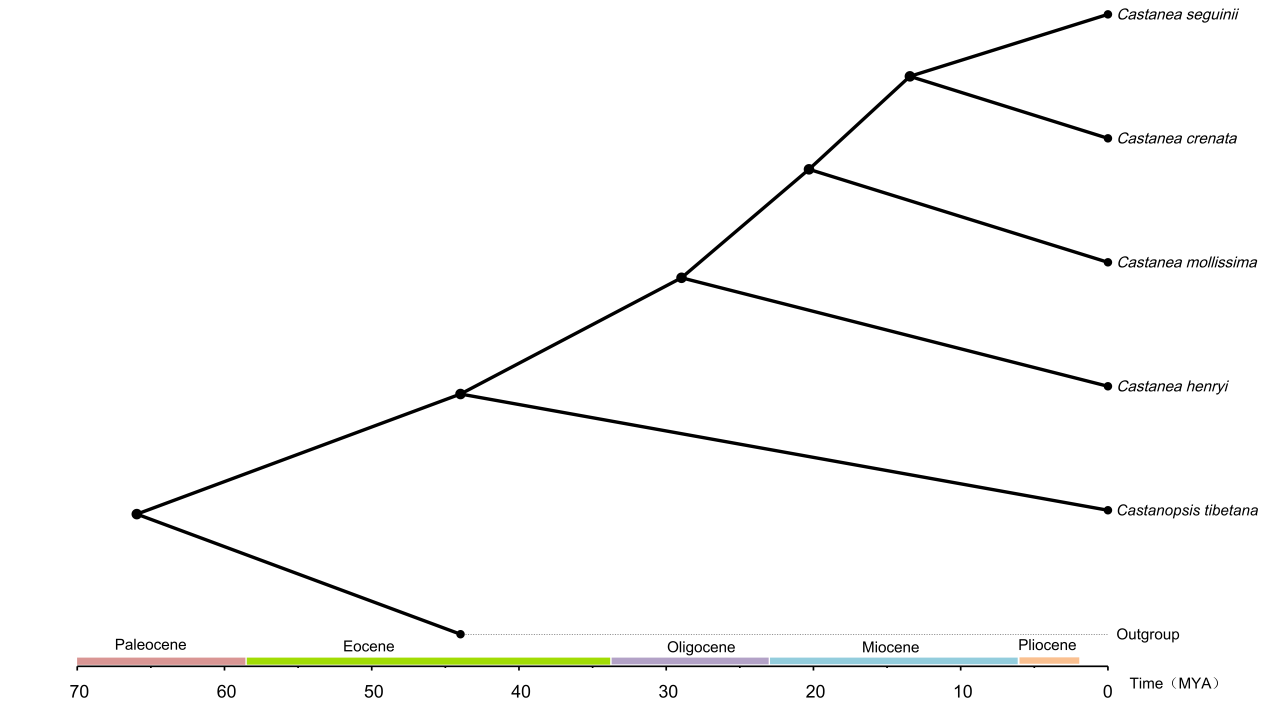


**Fig. S7** The divergence time of each species using 774 single-copy orthologous genes in East Asia.

**
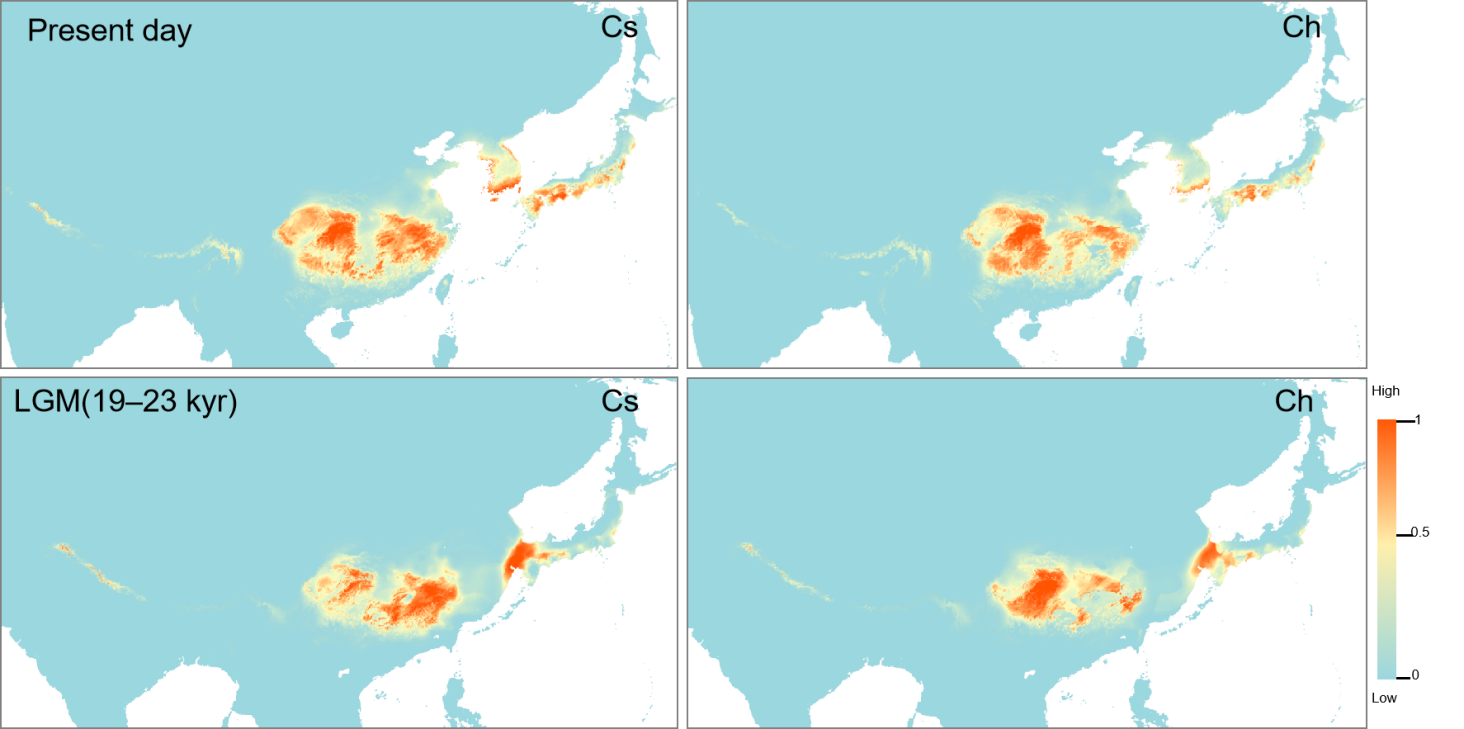
**

**Fig. S8** Prediction of the climate suitability of *C. seguinii* (Cs), *C. henryi* (Ch) based on the Maxent model, the color scale shows the suitability rating. During the LGM period, Cs and Ch were affected by extreme climate conditions, resulting in a lower living space compared to their current adaptation range. The Qinba-Wushan Mountains and the border between Chinese Mainland and the Japanese archipelago were the best adaptation areas, which indicated that there was extensive gene flow between Japanese chestnut and chestnut plants in Chinese Mainland in history.


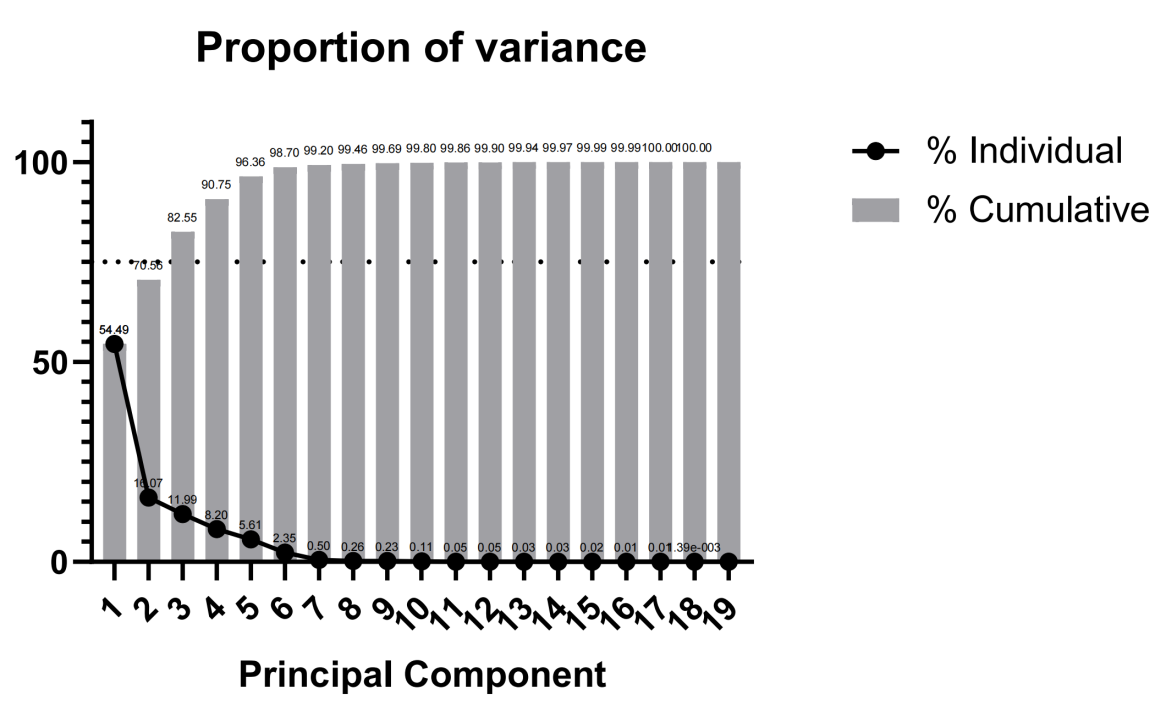


**Fig. S9** The principal component analysis of 19 EVs, the interpretation rate of PC1 reached 54.49%.


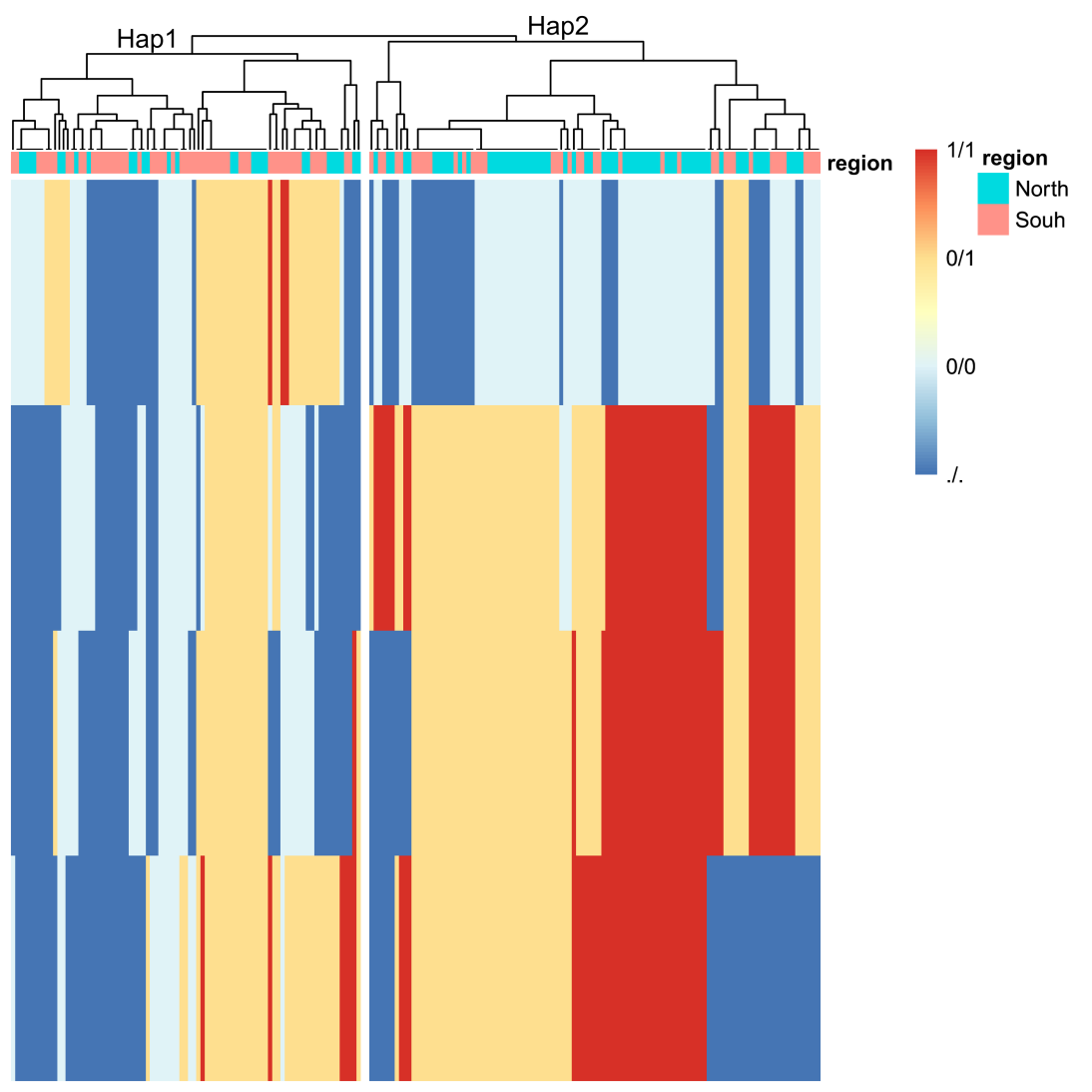


**Fig. S10** Distribution of Two Haplotypes of *CmLPA3* in North South Region.


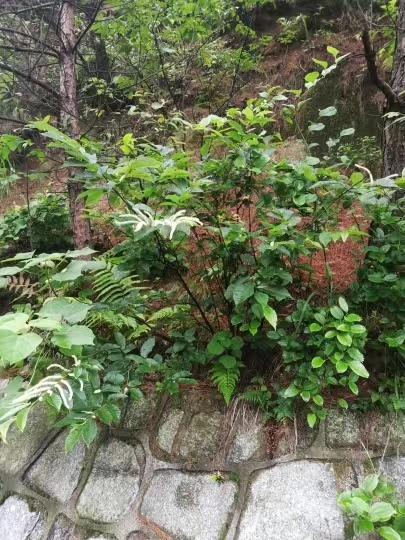

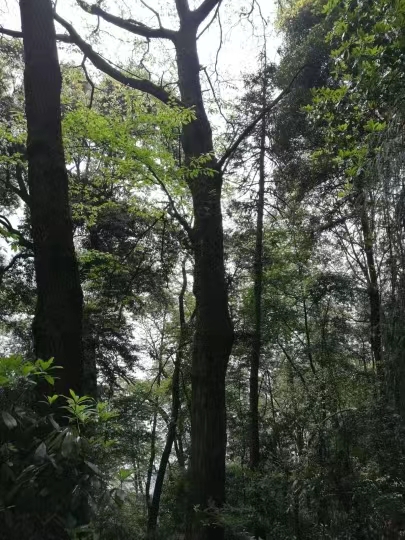


**b**

**a**

**Fig. S11** Legend of Seguin chestnut resources of different ecotype. a classic Seguin chestnut (shrub) in Yizhang County, Chenzhou City b tall Seguin chestnut (tree) inShennongjia, Yichang City.


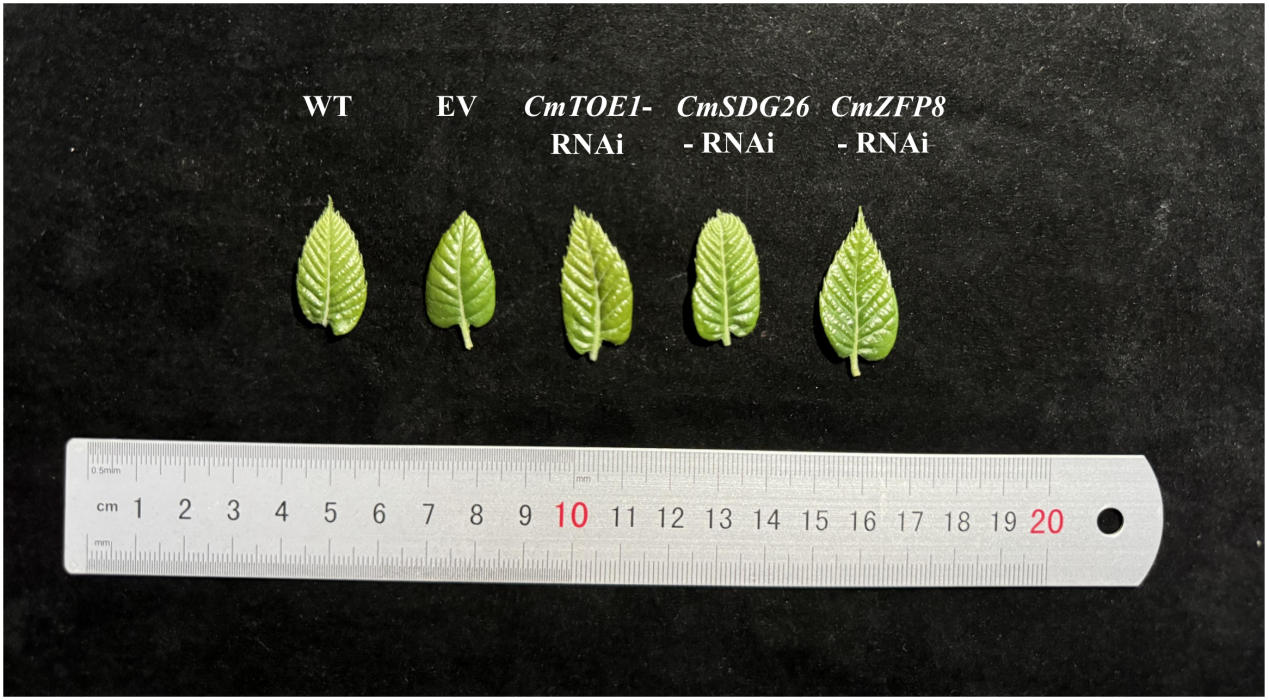


**Fig. S12** The leaves state of *CmTOE1*-RNAi, *CmSDG26*-RNAi and *CmZFP8*-RNAi transgenic tall Seguin chestnut.

**Table. S8** Related primer information of *CmTOE1*, *CmSDG26* and *CmZFP8*

| Primer | Sequence (5' to 3') | Tm |
| --- | --- | --- |
| CmTOE1-RNAi-F | GGGGACAAGTTTGTACAAAAAAGCAGGCTTCGACCTATGGAAGCATCTGAGCAT | 56.7 |
| CmTOE1-RNAi-R | GGGGACCACTTTGTACAAGAAAGCTGGGTATGTTGGTGATGTAAGTGATTGTTGG | 55.7ºC |
| CmSDG26-RNAi-F | GGGGACAAGTTTGTACAAAAAAGCAGGCTTC TCTACGATTCTGCCGAGGAT | 55.2ºC |
| CmSDG26-RNAi-R | GGGGACCACTTTGTACAAGAAAGCTGGGTA GCTTCTGCGACGAGTGTT | 55.5ºC |
| CmZFP8-RNAi-F | GGGGACAAGTTTGTACAAAAAAGCAGGCTTCCAACAACAGTGAAAACGGTG | 52.6ºC |
| CmZFP8-RNAi-R | GGGGACCACTTTGTACAAGAAAGCTGGGTACCATAATACCTGGCTGTGTT | 52.3ºC |
| CmToE1-qPCR_F: | ACGATGACTCTACTTCCAACTAC | 53.7 ºC |
| CmToE1-qPCR_R: | CGTCCTCCTCTTCCTCTTC | 54.1ºC |
| CmSDG26-qPCR_F | TCGTCGCAGAAGCAAGTT | 54.6ºC |
| CmSDG26-qPCR_-R: | CAATGGCAGGTCGTATCTCA | 54.5ºC |
| CmZFP8-qPCR_F: | TGGACAAGACGAGTGATAGAGA | 54.7ºC |
| CmZFP8-qPCR_R: | CGGATTCATCTGTGGTGGAA | 54.8ºC |
| actin-F | TTGACTATGAGCAGGAACTT | 51ºC |
| actin-R | TTGTAGGTGGTCTCGTGAAT | 53.3 ºC |
| 277KanaF | AGAGAGATAGATTTGTAGAGAGA | 49.5ºC |
| 277KanaR | TCAACTGTCACTGTAATACG | 49.5ºC |


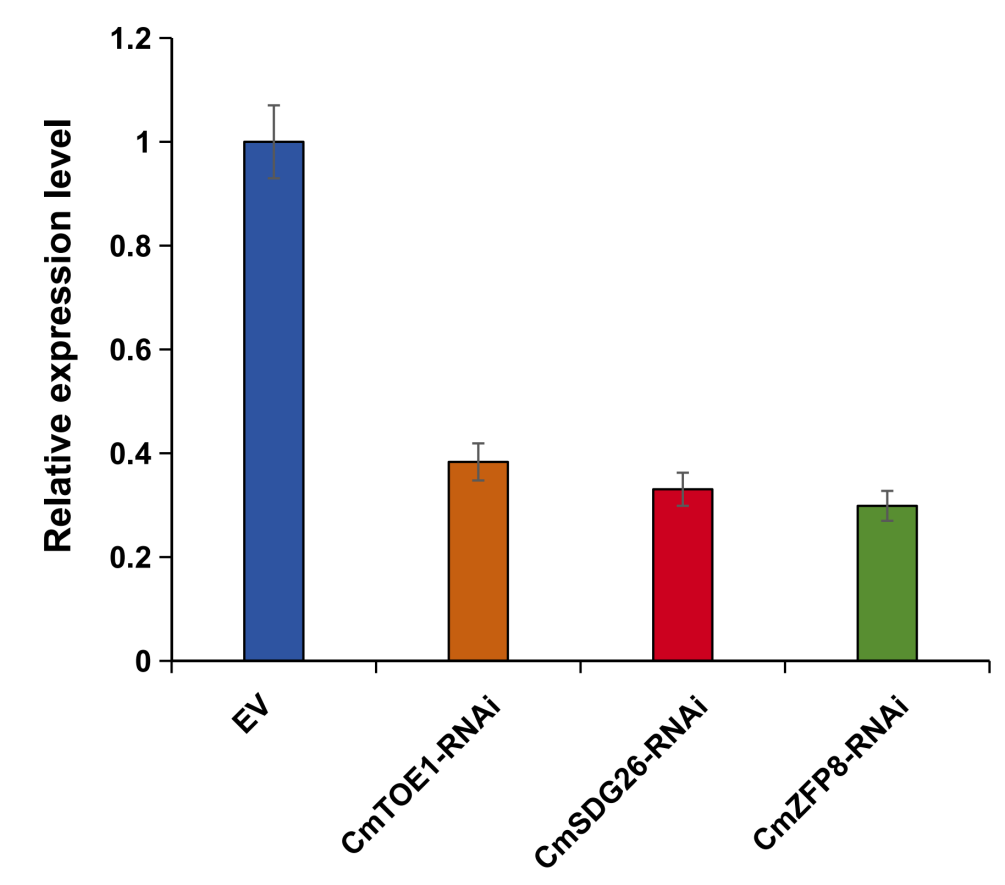


**Fig. S13** The relative expression level of *CmTOE1*-RNAi, *CmSDG26*-RNAi and *CmZFP8*-RNAi transgenic tall Seguin chestnut.


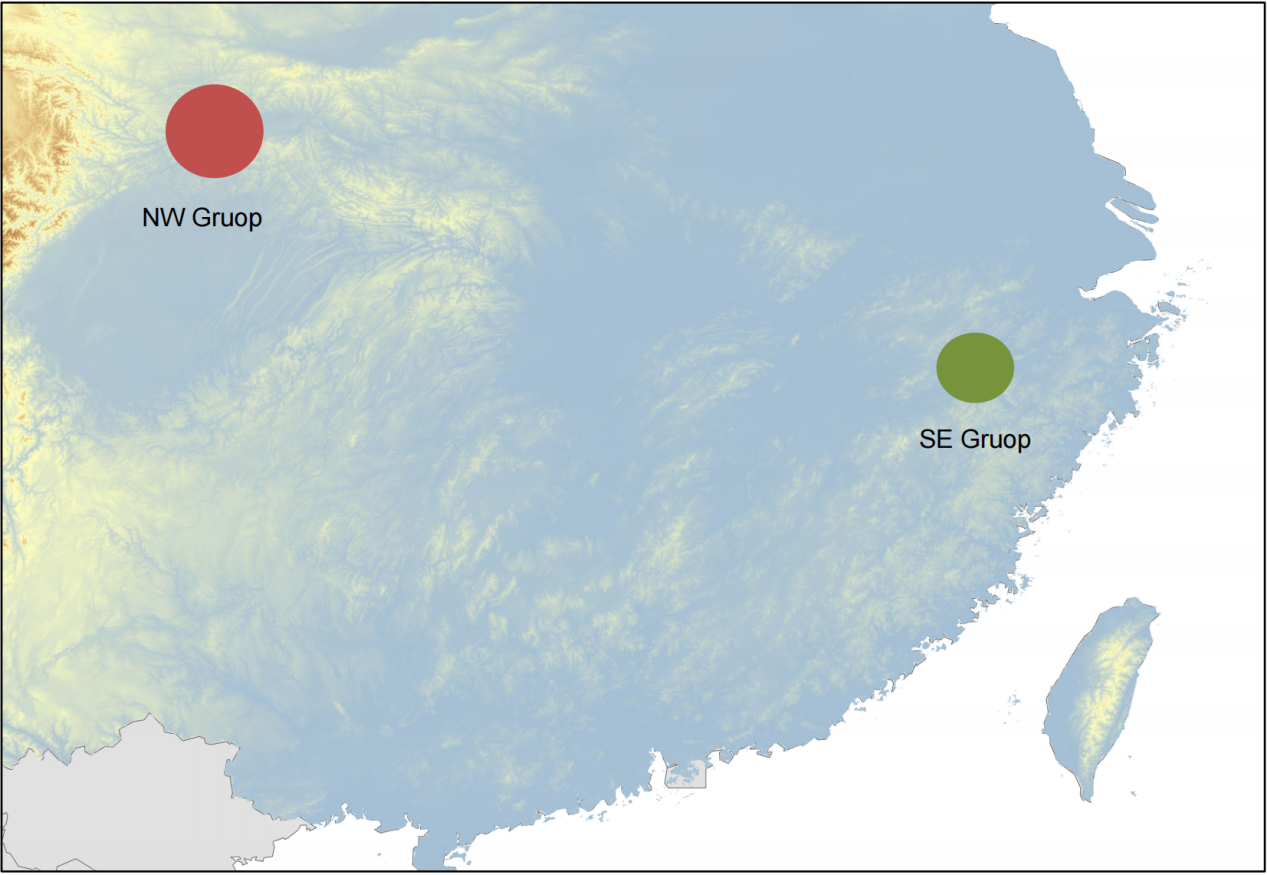


**Fig. S14** Geographic distribution of NW and SE groups.


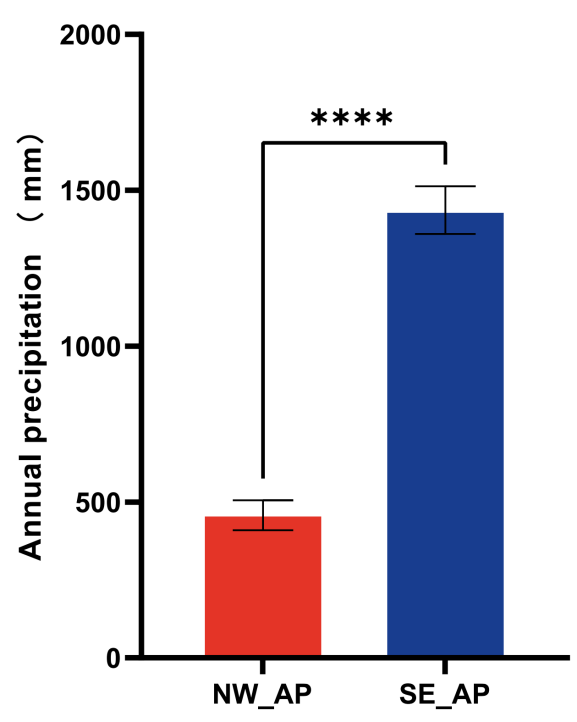


**Fig. S15** Difference in precipitation between the northwest group and the southeast group.


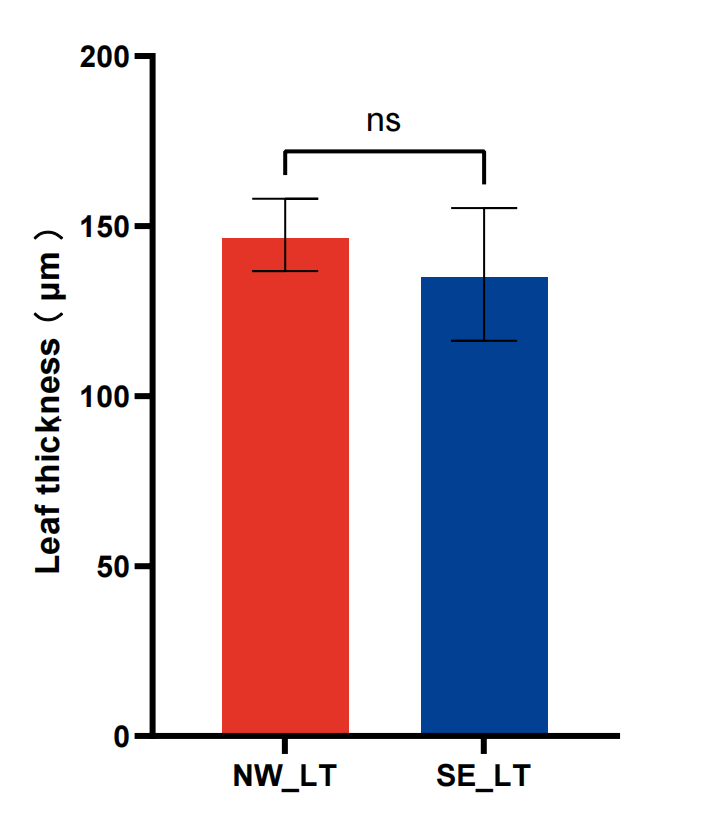


**Fig. S16** Analysis of variance of leaf thickness for mature leaves in the NW group and the SE group.

**
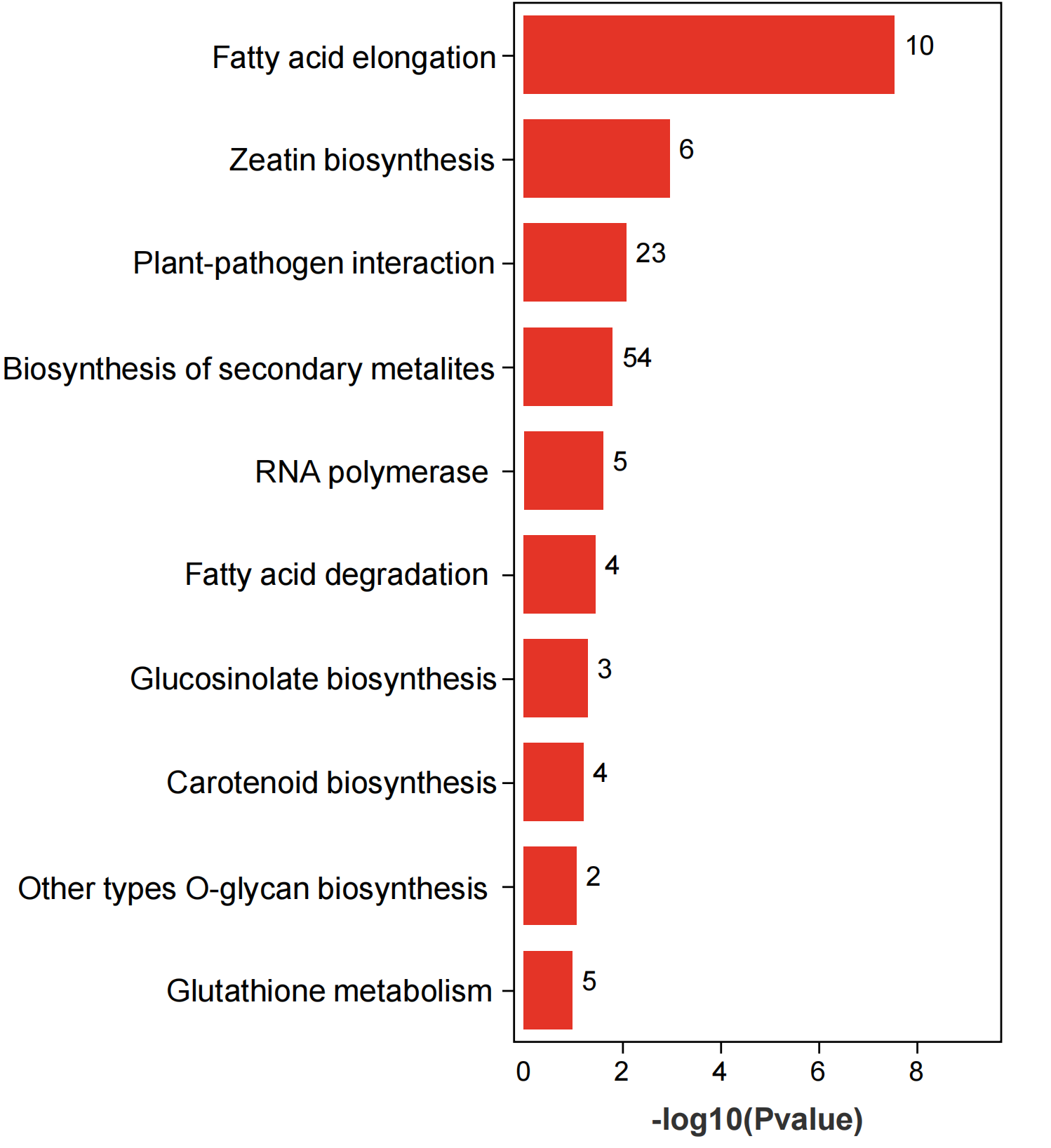
**

**Fig. S17** KEGG enrichment analysis of selected genes in SE groups.


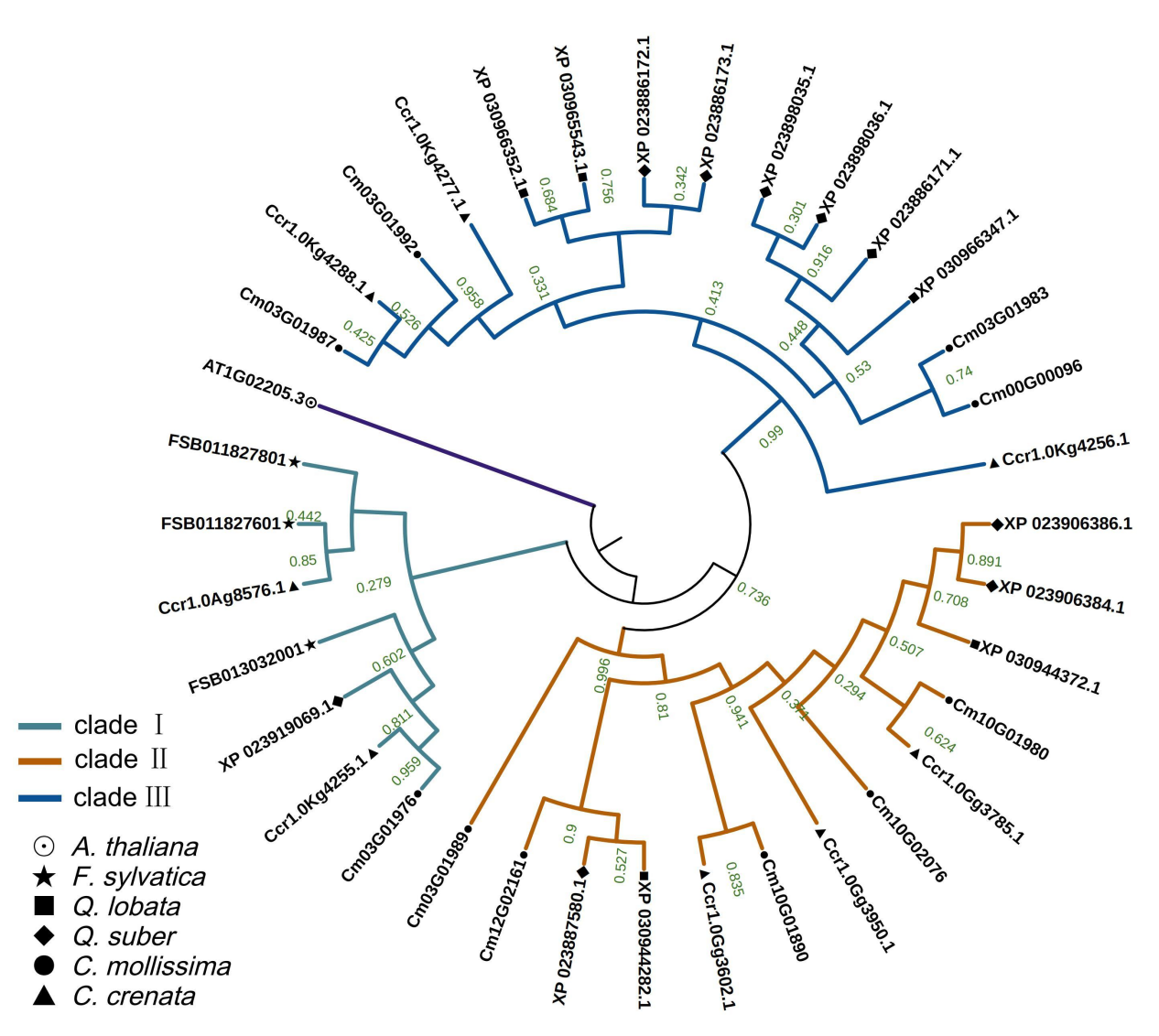


**Fig. S18** Clustering of *CER1* family genes across species, with more *CmCER1* members classified into three categories in Chinese chestnut.


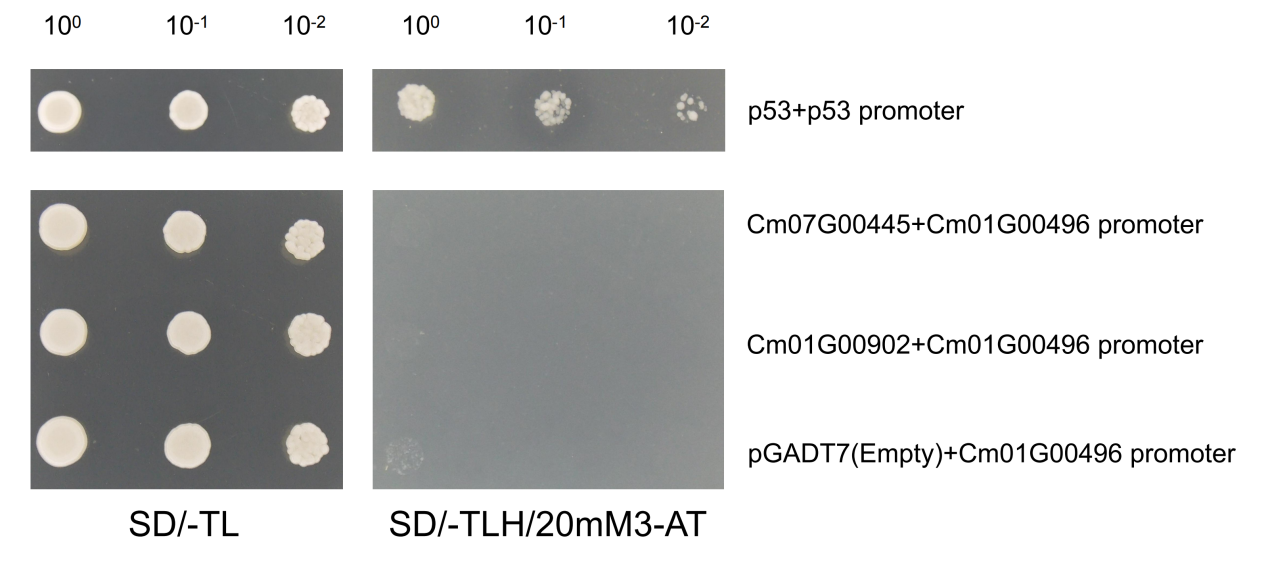


**Fig. S19** Verification of no interaction *CmSAGL1.1* (Cm07G00445) and *CmCER3* (Cm01G00496), *CmSAGL1.2* (Cm01G00902) and *CmCER3* (Cm01G00496) using a yeast one-hybrid assay.
